# Determining properties of human‐induced pluripotent stem cell‐derived cardiomyocytes using spatially resolved electromechanical metrics

**DOI:** 10.1113/JP287275

**Published:** 2025-02-17

**Authors:** Karoline Horgmo Jæger, Verena Charwat, Kevin E. Healy, Samuel Wall, Aslak Tveito

**Affiliations:** ^1^ Department of Computational Physiology Simula Research Laboratory Oslo Norway; ^2^ Johannes Kepler University Linz Austria; ^3^ Organos Inc Berkeley California USA; ^4^ Department of Material Science and Engineering University of California Berkeley California USA; ^5^ Department of Bioengineering University of California Berkeley California USA

**Keywords:** cardiac electrophysiology, cell based modeling, computer modelling, drug effect identification, human induced pluripotent stem cell‐derived cardiomyocytes

## Abstract

**Abstract:**

Human‐induced pluripotent stem cell‐derived cardiomyocytes (hiPSC‐CMs) are increasingly important in preclinical drug assessments, particularly for identifying potential cardiotoxicity. In this study, we utilize data from microphysiological systems of hiPSC‐CMs to evaluate cellular characteristics, such as action potential duration, beat rate, conduction velocity and mechanical displacement. Based on these data, high‐fidelity mathematical models facilitate precise assessments of critical biophysical parameters of the cells, including membrane ion channel conductances, cross‐bridge cycle transition rates and cell‐to‐cell conductance. We emphasize the distinction between synchronized transients and travelling waves, highlighting their implications for deducing the biophysical properties of hiPSC‐CMs. In this study, we analyse the effects of the drug compounds flecainide, quinidine, nifedipine, verapamil, blebbistatin and omecamtiv. Our findings show that for drug‐induced changes in parameters describing membrane currents and contractile machinery close to ranges reported in the literature, the computed biomarkers align well with measured biomarkers. This study is the first to apply spatially resolved, cell‐based models to identify drug effects through measurements of transmembrane potential and mechanical displacement, marking a significant step forward in using computational models for evaluating drug safety and offering a new approach to early identification of adverse drug reactions.

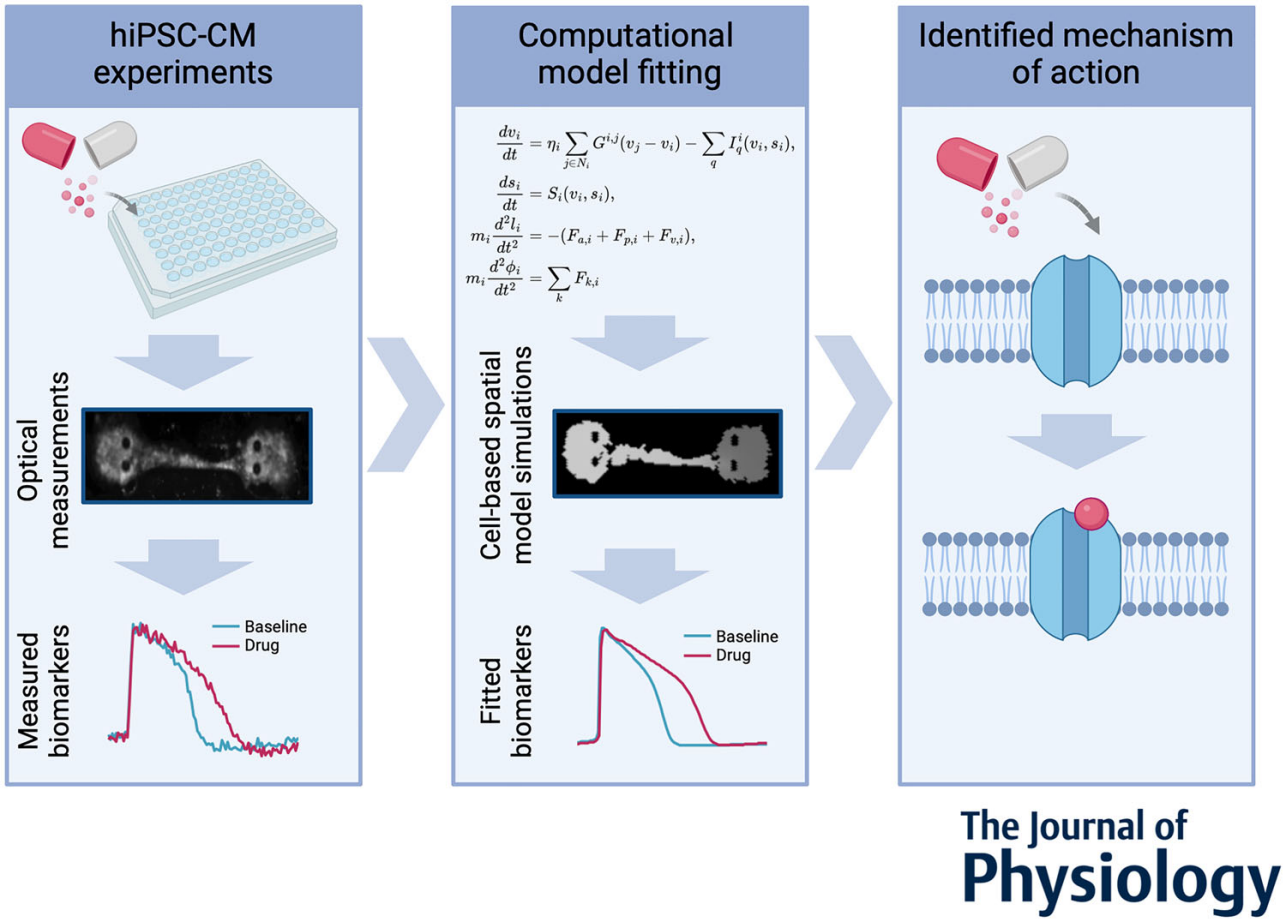

**Key points:**

- Optical measurements of human‐induced pluripotent stem cell‐derived cardiomyocytes (hiPSC‐CMs) present significant opportunities to advance understanding of how human heart cells function and interact.
- Although direct optical measurements yield valuable biomarkers, they fall short of revealing underlying biophysical properties, for example, how novel drug compounds perturb the ion channels.
- Drug properties are best understood through computational models that capture cell dynamics based on physical laws.
- Traditionally, data and models have been averaged over all cells in cell collections, thus overlooking spatiotemporal waves.
- Here, we use recently developed cell‐based models, representing spatial dynamics including cell‐to‐cell electrical and mechanical coupling, to determine biophysical properties of collections of hiPSC‐CMs.

## Graphical abstract

**Abstract figure legend** In this study, we use human‐induced pluripotent stem cell‐derived cardiomyocyte (hiPSC‐CM) experiments and computational modelling to identify the mechanism of action of drug compounds. In the hiPSC‐CM experiments, optical measurements of cell collections are recorded in the baseline case and after drug exposure. From these images, electromechanical biomarkers like action potential duration, beat rate, conduction velocity and mechanical displacement are evaluated. These biomarkers are then used to fit the biophysical parameters of a cell‐based spatially resolved computational model of collections of hiPSC‐CMs. Parameters are identified such that simulations of the parameterized computational model replicate the biomarkers observed in the experiments. The drug's mechanism of action in terms of alterations in ion channel function or cross‐bridge cycle transitions is subsequently revealed by comparing the biophysical parameters in the baseline and drug‐exposed cases.

## Introduction

The use of animal testing for investigating properties of new drug compounds remains widespread (Mukherjee et al., 2022), but significant challenges reduce the accuracy of these models (Ingber, 2022; Loewa et al., 2023). As noted by Ingber (2022), concerns about the usefulness of animal models extend even to basic research: *The failure of animal models to predict therapeutic responses in humans is a major problem that also brings into question their use for basic research*. A promising alternative is the use of cells derived from human‐induced pluripotent stem cells (hiPSCs) organized in microphysiological systems (MPSs), often referred to as Organs‐On‐Chips (Ingber, 2022; Loewa et al., 2023; Mummery, 2018; Stein et al., 2021). For example, hiPSC‐derived cardiomyocytes (hiPSC‐CMs) can be used to study the effect of new drug compounds on human heart cells. A substantial advantage of hiPSC‐CMs compared to animal models is that these cells are human, which can lead to more accurate predictions of human responses. Additionally, hiPSC‐CMs enable personalized medicine by allowing drugs to be tested on cells carrying the specific genetic material of an individual patient. However, it must be noted that these cells are still immature. Differences between adult and immature cells are apparent in biomarkers, reflecting concerted properties of large numbers of cells with an even larger number of active constituent proteins.

The application of hiPSC‐CMs in understanding drug effects and reducing the substantial costs associated with drug development requires reliable MPSs. These MPSs are essential for measuring biomarkers such as action potential duration (APD), mechanical displacement amplitude (DA) due to cell contraction, conduction velocity (CV), beat rate (BR) and more. The data used in this study are sourced from the systems described in Charrez et al. (2021), Charwat et al. (2022) and Huebsch et al. (2022).

Although direct measurements of biomarkers such as APD, BR, DA and CV provide valuable data, they offer limited insight into the underlying physical properties and intercellular coupling mechanisms of hiPSC‐CMs. To transform these raw measurements into meaningful biophysical quantities, it is essential to employ mathematical models. These models allow us to deduce critical cellular properties. Consequently, the mathematical models not only enhance the interpretative power of the MPS measurements but also significantly augment their scientific value, enabling a deeper understanding of cellular dynamics and interactions. The use of mathematical models in electrophysiology is carefully reviewed in Trayanova et al. (2024) and Niederer et al. (2019), and a basic introduction to the models and their numerical solution is provided in Jæger and Tveito (2023a).

In this study, we demonstrate how biomarker data from MPSs can be utilized in models to infer biophysical cell properties, including maximum membrane channel conductances, cross‐bridge cycle transition rates and the strength of gap junction coupling in terms of cell‐to‐cell conductances. Enabling this inversion from biomarkers to biophysical understanding is essential, as it facilitates the identification of the biophysical changes caused by, for example, the applications of drugs. Data on biomarkers such as APD, DA and BR can be used to determine the biophysical parameters defining the mathematical model. This kind of inversion, from observation to model, is done routinely in the field; see, for example, Barral et al. (2022), Mirams et al. (2016), Paci et al. (2020), Paci et al. (2018), Soepriatna et al. (2023), Tveito et al. (2018) and Whittaker et al. (2020). Albeit used routinely, the problem of determining the correct parameters to match a set of biomarkers is highly non‐trivial and can be confounded by issues of non‐uniqueness, in which different parameters yield the same or very similar resulting biomarkers (Dokos & Lovell, 2004; Groenendaal et al., 2015; Johnstone et al., 2016; Jæger et al., 2019; Marder & Taylor, 2011; Whittaker et al., 2020). However, results are important as not only do they help infer the processes that are occurring in the cell, but biophysical parameter identification also allows for synthetic translation from properties of the hiPSC‐CMs to corresponding properties of adult human cardiomyocytes (Gong & Sobie, 2018; Tveito et al., 2020, 2018).

One characteristic feature of hiPSC‐CMs is that, like fetal cells, they often function as pacemaker cells, in the sense that they beat rhythmically without external stimulation. For pacemaker cells, the data obtained in an MPS are usually synchronized throughout the chip. When the cells are synchronized, there are no spatial waves in the chip, and therefore the dynamics of the cells can be modelled without taking spatial gradient into account; see, for example, Bartolucci et al. (2020), Botti et al. (2024), Forouzandehmehr et al. (2021), Jæger, Charwat et al. (2021), Kernik et al. (2019), Paci et al. (2013) and Paci et al. (2020).

Although often acting as synchronized pacemaker cells, hiPSC‐CMs can also function as excitable tissue, setting off excitation waves that travel across the chip. This provides additional information, as the CV can be added to the list of observable biomarkers. Of particular interest is measuring how new drug compounds affect the entire vector of biomarkers, and using the data to specify the effects of the drugs on the biophysical properties of the cells. In the case of travelling waves, spatially resolved mathematical models must be applied for the calibration of the model.

For data from hiPSC‐CMs, calibration based on spatially resolved models has been much less studied than the case of synchronized data. The problem has been investigated using the classical bidomain model (see, e.g., Franzone et al., 2014), utilizing the extracellular potential measured by multielectrode arrays; see, for example, Abbate et al. (2018), Bouyssier and Zemzemi (2017), Jæger, Charwat et al. (2021), Raphel et al. (2017), Raphel et al. (2019) and Tixier et al. (2018), but the bidomain model is not ideally suited for data from an MPS for several reasons. First, the chip typically contains cells unevenly distributed, often with substantial areas not covered by cells. A model should accurately reflect this distribution, as illustrated in Figs 5 and 6. Second, the bidomain model fundamentally assumes that the intracellular (I) space, the cell membrane (M) and the extracellular (E) space are all present everywhere. Although this assumption is clearly incorrect, the bidomain model has nonetheless served the computational community well for decades by providing a reasonably accurate model on the millimetre scale (see Jæger, Edwards, Giles et al., 2021). However, given that cells are at the micrometre scale, more accurate models like the EMI model should be applied. For details on model formulation, numerical solutions and applications, see de Souza et al. (2024), Benedusi et al. (2024), Huynh et al. (2023), Jæger and Tveito (2021), Jæger and Tveito (2022), Telle et al. (2023) and Tveito et al. (2017). The EMI model accurately represents individual cells and distinguish between the E, M and I computational domains.

**Figure 1:**
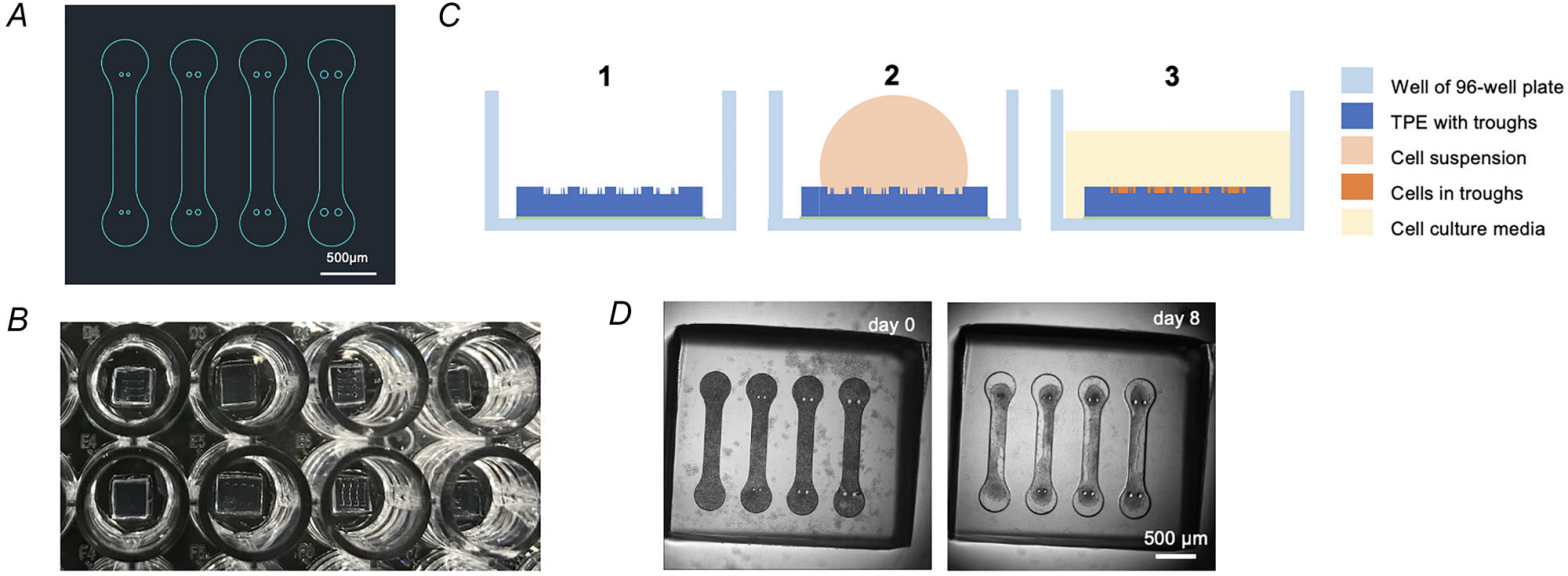
Open‐well cardiac microtissues (Samples C1–C6) (*A*) TPE trough design, (*B*) TPE cut‐outs (approx. 2.5 × 3.5 mm^2^) placed in a 96‐well plate and (*C*) schematic drawing (not to scale) of a TPE sample in a 96‐well plate (1). For cell loading, a drop of cell suspension is added covering the troughs (2). After cell packing into the troughs, fresh culture media are added (3). (*D*) CM1 cell loading (Day 0, left) and tissue formation (Day 8, right).

**Figure 2:**
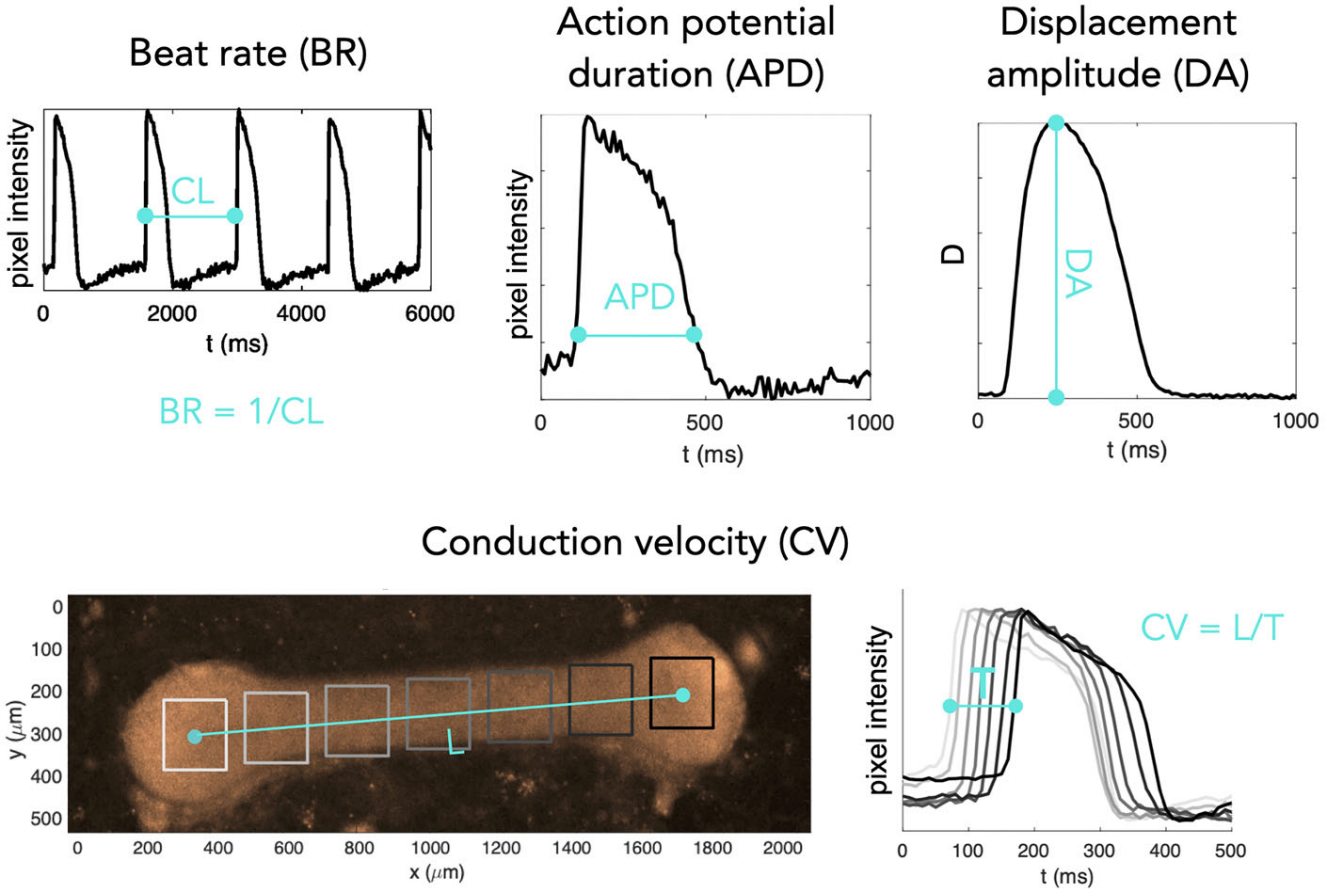
Illustrations of the considered biomarkers. The beat rate (BR), the action potential duration (APD) and the conduction velocity (CV) are computed from recordings of voltage sensitive dyes, and the displacement amplitude (DA) is computed from brightfield images as described in eqn (1).

**Figure 3:**
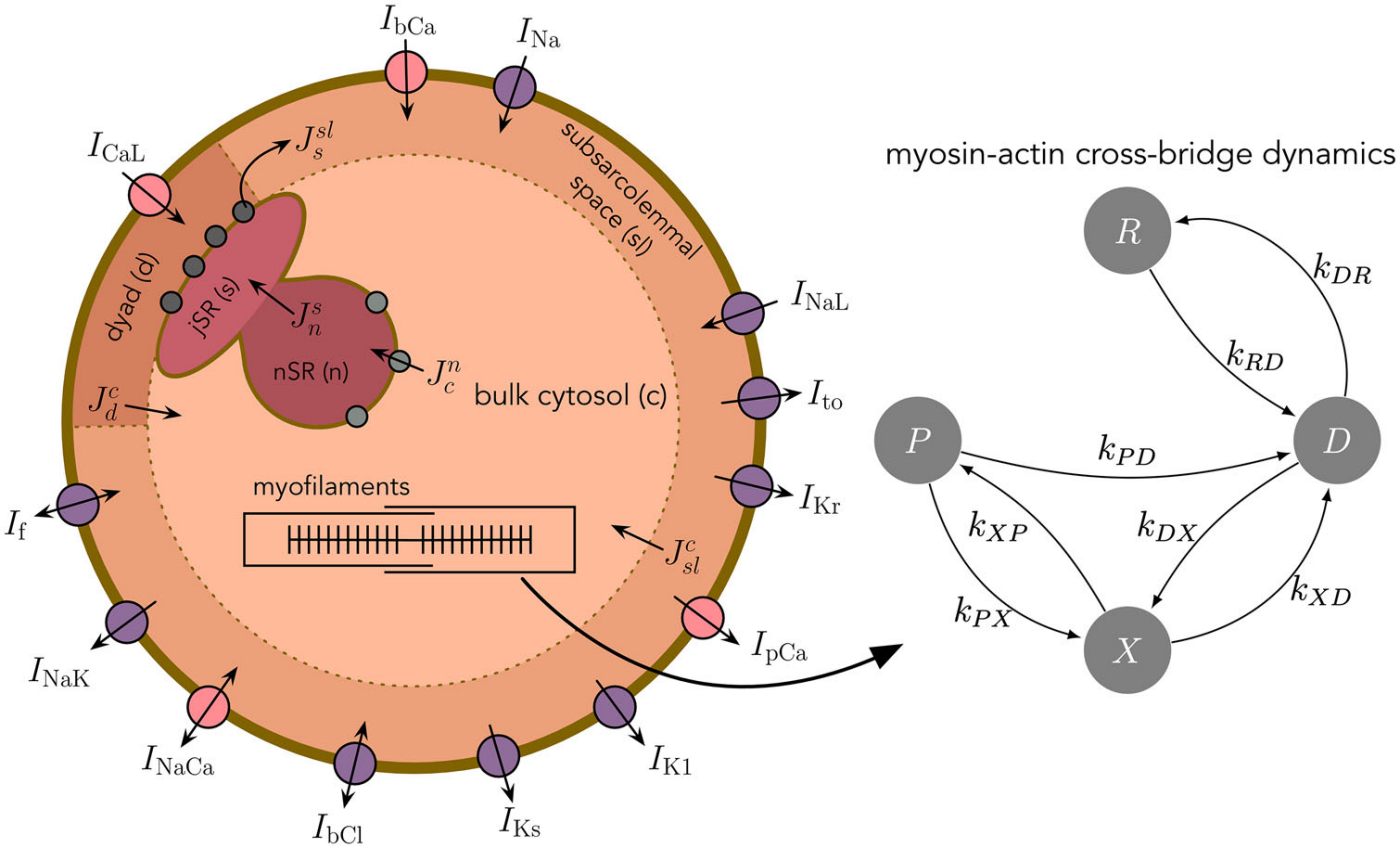
Illustration of components of the applied single‐cell model. The left panel illustrates the transmembrane currents and the intracellular calcium fluxes, in addition to the five considered intracellular compartments. The right panel illustrates the Markov model used to represent the myosin–actin cross‐bridge dynamics. This Markov model has four states, the non‐permissive resting state (R), the permissive detached state (D), the cross‐bridge pre power stroke (X) and the cross‐bridge post power stoke state (P).

**Figure 4:**
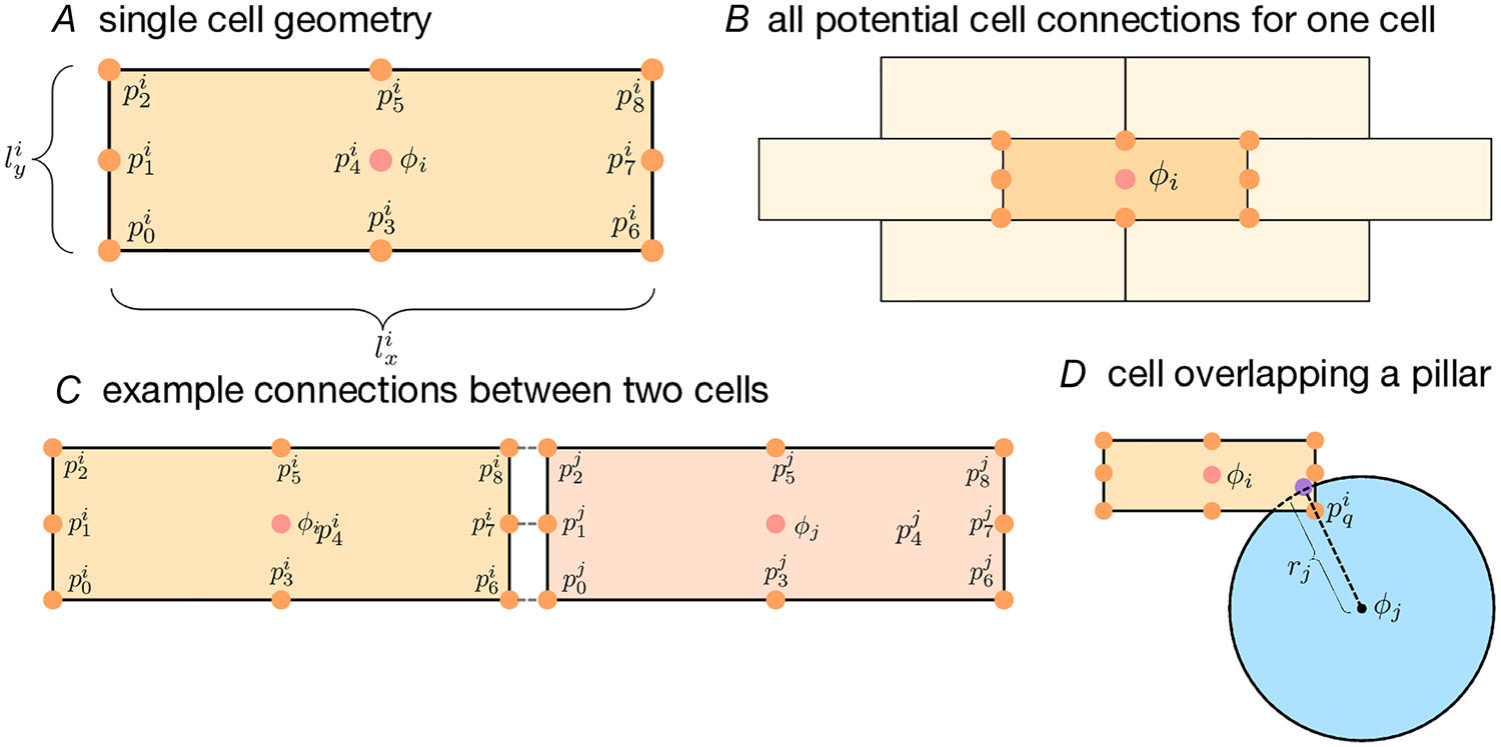
Illustrations of the cell geometry and possible cell connections in the spatial cell collection model The centre point of cell i is denoted by $\phi_{i}=p_{4}^{i}$ and the possible connection points are denoted by $p_{q}^{i}$, for q=0,1,2,3,5,6,7,8.

**Figure 5:**
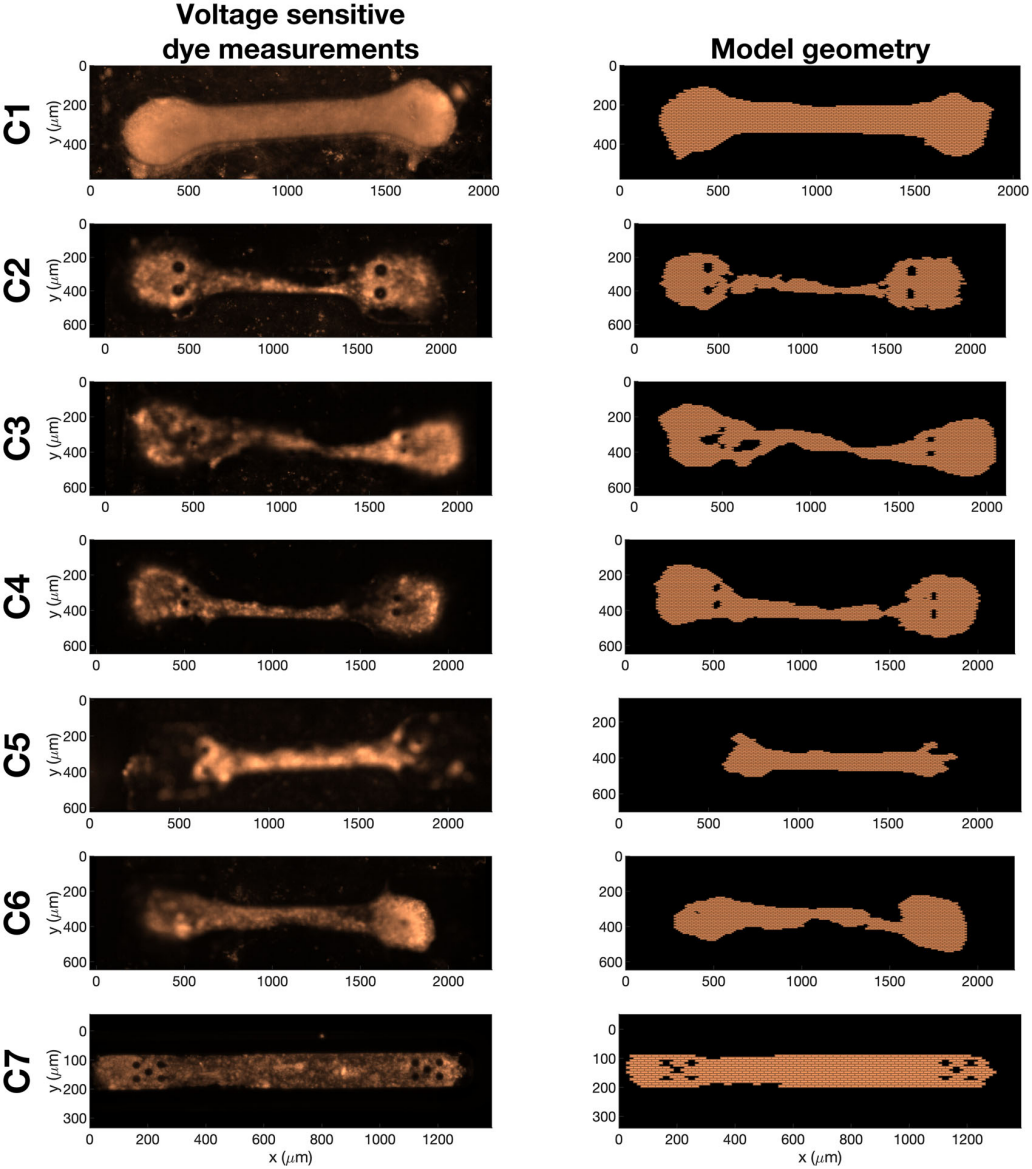
Illustration of the seven chips (i.e. cell collections) C1–C7 considered in this study. The left panel shows the average (over time) of the optical measurements using voltage sensitive dyes for the baseline case with no drug present. The right panel shows the geometry used in the computational model for each of the different cell collections.

**Figure 6:**
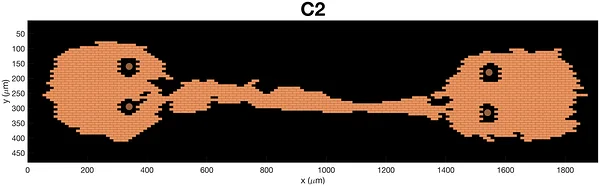
More detailed illustration of the model cell distribution in Chip C2. The brown round objects are pillars, and the orange rectangles are hiPSC‐CMs.

It was recently demonstrated that a model based on representing cardiac tissue in terms of individual cells as the computational unit is very efficient and offers significant modelling flexibility (see Jæger & Tveito, 2023b, 2023c, 2024; Ricci et al., 2024). The concept of using individual cells as the computational unit has been explored previously (see, e.g., Kucera et al., 2002; Kuijpers et al., 2005), but the use of such models was overshadowed by homogenized models like the bidomain and monodomain models due to their computational and implementational efficiency. However, with spatial resolutions approaching the level of individual cells, cell‐based models become competitive (see Jæger et al., 2024b). The cell‐based models derived in Jæger and Tveito (2023b), Jæger and Tveito (2023c) accurately represent the electrochemical waves traversing cardiomyocytes. Here, we will extend this concept to account for the mechanical displacements of the cells caused by the contraction of each cell. This effect is easily observed in MPS data, and we will show that displacement data can be used to determine the biophysical parameters characterizing the contractile apparatus of the cells.

## Methods

Our main objective is to demonstrate how data from an MPS can be interpreted to reveal biophysical parameters characterizing hiPSC‐CMs. In this section, we describe the applied experimental methods and the process of obtaining biomarkers from MPSs. In addition, we explain how a new mathematical model can transform these biomarkers into meaningful biophysical parameters.

### Imaging voltage and displacement of hiPSC‐CMs in MPSs

In this section, we describe the experimental set‐up used to obtain images of voltage and displacement of hiPSC‐CMs in MPSs.

#### Sample preparation

All samples consisted of human‐induced pluripotent stem cell‐derived cardiomyocytes (hiPSC‐CMs) cultured in small, geometrically defined microchambers. The specific device and cell culture parameters varied between the samples, and details are outlined below and in Table 1. Samples C1–C6 (see overview in Fig. 5) were cultured in 96‐well plates with custom inserts cut out from injection moulded thermoplastic (Topas, cyclic olefin copolymer [COC] TPE E140) provided by Stratec, Austria. The TPE discs featured custom troughs of 150 μm depth (see Fig. 1 *A* for geometry). Each trough had two anchor pillars of varying diameter (30–60 μm) on either side of the chamber. The moulded TPE plate features were cut to size (approx. 2.5 × 3.5 mm^2^, four troughs per sample) and glued to the centre of each well using a drop (about 20 µL) of liquid PDMS that was cured overnight at 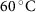 (Fig. 1 *B*). The final assembled devices were sterilized under UV light followed by washing steps with 70% ethanol and PBS (21‐031‐CV, Corning) to remove air bubbles and prime for cell loading. Sample C7 was prepared in a microfluidic device made from PDMS on a glass slide, and a detailed method description has been published (4‐plex device, Charwat et al., 2022).

**Table 1:** Summary of samples, cell composition, tissue age, substrate, format and drug used

Sample	Cells	Tissue age	Substrate	Format	
C1	100% WTC‐CM	2 days	TPE	Static	Control
C2	100% CM1	12 days	TPE	Static	Flecainide
C3	100% CM1	8 days	TPE	Static	Quinidine
C4	100% CM1	8 days	TPE	Static	Nifedipine
C5	100% CM1	11 days	TPE	Static	Verapamil
C6	100% CM1	11 days	TPE	Static	Blebbistatin
C7	80% WTC‐CM	15 days	Microfluidic	Perfused	Omecamtiv
	20% HCF				

#### Cell loading and tissue formation

Cardiomyocytes from two different sources were used: in‐house differentiated cardiomyocytes from the hiPSC‐line Wild Type C (WTC; #GM25256, Coriell Institute) (see Charwat et al., 2022 for culture details) and commercially available hiPSC‐CM Donor: 01434 (R1007, Fujifilm Cellular Dynamics). Below we refer to those cells as *WTC‐CM* or *CM1*, respectively. For cell loading in C1–C6, the PBS was aspirated and a small amount of residual liquid kept the troughs air bubble free (Fig. 1 *C*, Step 1). Then, a 10 µL drop of concentrated single‐cell suspension was pipetted onto the TPE sample covering all four troughs (Fig. 1 *C*, Step 2). A total of 100,000 (CM1) or 200,000 (WTC) cells were used for each well. The cells were allowed to sediment for 10 min. Then the plate was centrifuged for 3 min at 300 rcf. This further packed the cells into the troughs and removed a majority of cells from the TPE plateau area. In a gentle rinsing step, more cells were washed off the plateau region. Finally, 100 µL culture media were added to each well. The well area around the TPE sample was used for gentle pipetting to prevent cell washout from the troughs (Fig. 1 *C*, Step 3). Onset of tissue formation and spontaneous beating was observed within 24–48 h and tissues could be used between Days 2 and 16 (Fig. 1 *D*). The anchor pillars helped to hold the tissue in place and supported the formation of a microfibre instead of condensation into a ball‐shaped spheroid. During tissue formation, the cells condensed and the final tissues covered only part of the trough area. Figures 1 *D* and 5 depict the variability in area coverage, tissue shapes and cell distribution. Final tissues covered between 60 and 100% of the original trough area and contained approximately 3000–10,000 cells.

For Sample C7, a mix of 20,000 cells in 3 µL media containing 80% WTC‐CM (Charwat et al., 2022, WTC cells) and 20% human cardiac fibroblasts (C‐12375, Promocell) was used for loading of the cell chamber. After 1‐day incubation in EB20 with rock inhibitor, the media were switched to fatty acid‐rich maturation media for 14 days (see Huebsch et al., 2022 or Charwat et al., 2022 for detailed media compositions). In contrast to the open well format, for the microfluidic sample, the cell chamber was geometrically defined in all three dimensions. Sample C7 was formed from a fully loading cell chamber, and the microtissue retained the shape defined by the chamber geometry, filling the space almost completely (see Fig. 5).

#### Drug exposure and video acquisition

On the day before the drug test, microtissues were stained with 500 nM of the voltage sensitive dye BeRST‐1 (08‐10003, Lumencor; Huang et al., 2015) overnight. Excess dye was washed out directly before the experiment. For drug experiments, the samples were placed inside the incubation chamber (5% CO_2_, 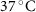, humidified) of an ImageXpress Micro (Molecular Devices) microscope. Sample C1 served as a no drug control. For drug dose‐escalation studies in Samples C2–C6, the volume per well was kept constant at 200 µL. Drug doses were prepared so that taking out 50 µL and adding 50 µL of the next higher dose yielded the desired concentration. Cell responses were recorded for each dose (see description of video acquisition below). For the perfused sample (C7), a pressure‐driven pump (M‐switch, Fluigent) and multiple inlet vials, each containing a different drug dose, were used and set to a constant flow rate of 11 µL/min (∼72 mbar). Every 40 min, the M‐switch valve switched to the next higher drug dose. For all samples, incubation time before recording was 20–40 min for each dose. All acquisition was performed on the ImageXpress Micro workstation. Videos using brightfield (10% light power, 10 ms) and Cy‐5 channels (2% laser power, 10 ms) were recorded in each well for about 6 s via a 4× objective with 2 × 2 binning and 80% × 80% field of view.

### From images to biomarkers

In this section, we describe how we compute relevant biomarkers from images of hiPSC‐CMs in MPSs.

#### Computing voltage traces from voltage sensitive dye images

Given image recordings of voltage sensitive dyes, voltage traces are computed by considering the average pixel intensity in some area of the cell collection. As will be explained in the Results section, in the case of synchronously beating cells, we take the average over the entire chip, whereas in the case of non‐synchronous cells (see, e.g., Fig. 7), local averages are computed in smaller areas of the cell collection. A typical voltage trace computed from voltage sensitive dye images is displayed in the upper left panel of Fig. 2.

**Figure 7:**
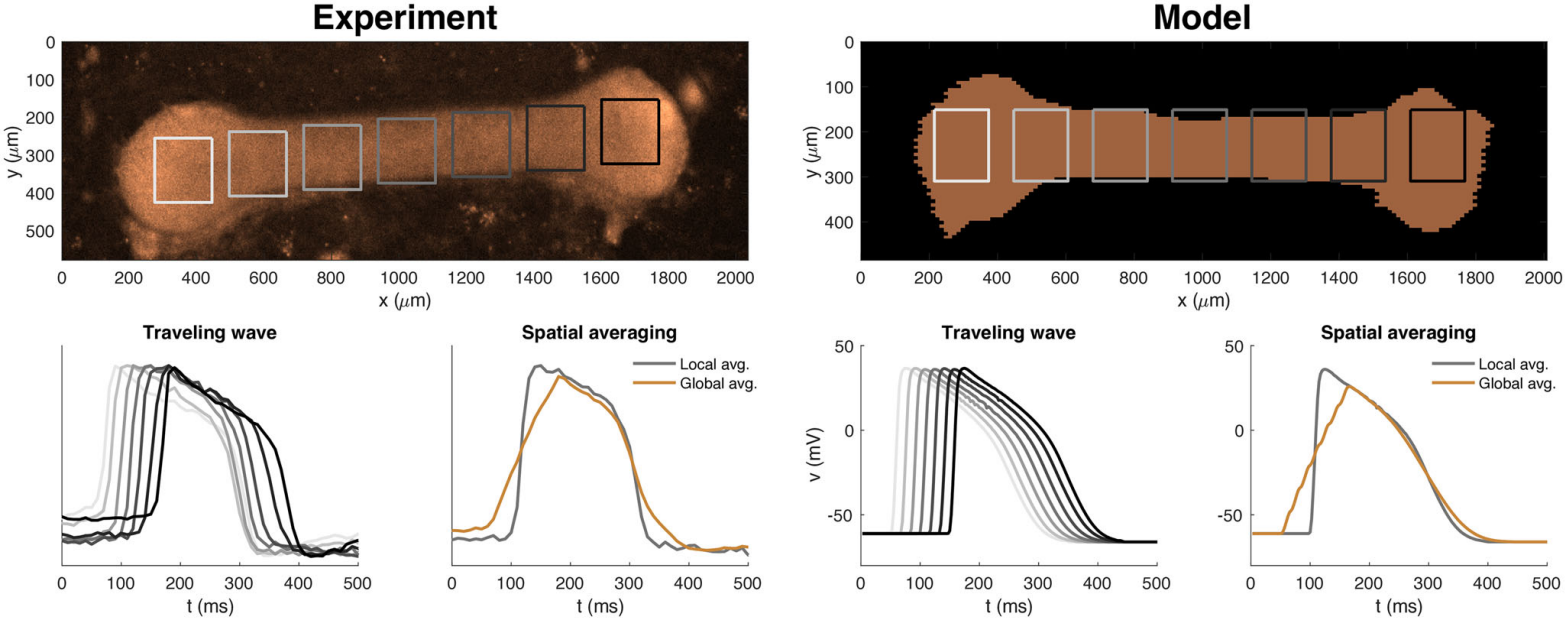
Illustration of a slow electrical wave travelling through Chip C1. The left panel shows optical measurements from an experiment, and the right panel shows the model solution. The lower first and third panels show the average transmembrane potential in seven local areas of the chip. The upper panel illustrates the local areas used to define the transmembrane potential in the lower panel. The second and fourth lower panels compare the action potential in the fourth local area (grey) to the average trace over all seven areas (orange).

#### Computing BR from voltage trace

From the voltage trace, we compute the BR by recording the time points when the pixel intensity is increasing and crossing the threshold value located halfway between the maximum and minimum values of the voltage trace (see upper left panel of Fig. 2). The distance in time between two such points is defined as the cycle length (CL), and the BR (in Hertz) is defined as BR = 1/$\bar{\mathrm{CL}}$, where $\bar{\mathrm{CL}}$ is the average cycle length (in seconds).

#### APD from voltage trace

The APD is computed from the voltage trace by recording, for each of the included action potentials, the time when the pixel intensity increases most rapidly, $t_{1}$, and the point in time, $t_{2}$, when the value is decreasing and crossing the threshold of 80% repolarization (i.e. $v_{th}=v_{\max}-0.8\times \left(v_{\max}-v_{\min}\right)$), where $v_{\max}$ and $v_{\min}$ are the maximum and minimum values of the pixel intensity (see upper middle panel of Fig. 2). The APD (in milliseconds) is defined as the distance in time between $t_{2}$ and $t_{1}$, and we use the median value over the recorded action potentials in the trace as the representative APD value. The choice of 80% reploarization aims to reduce the impact of random noise in the measurement data, which would be more pronounced at 90% repolarization.

#### Computing CV from local voltage traces

To compute the CV, we record average voltage traces over local areas of the cell collection as illustrated in the lower left panel of Fig. 2. The local traces during one action potential are illustrated in the lower right panel. Here, the colour of the trace corresponds to the colour of the local average windows displayed in the left panel. The CV (in cm/s) is defined as CV = *L*/*T*, where *L* is the distance (in centimetres) between the centres of the leftmost and rightmost local windows, and *T* is the distance (in seconds) between when the voltage traces from the leftmost and rightmost local windows first reach a pixel intensity that is halfway between the maximum and the minimum values (see lower panel of Fig. 2).

#### Computing displacement traces from brightfield images

To compute displacement traces from brightfield images, we use a simple measure of changed brightfield intensity:

(1) $$D\left(t_{n}\right)=\frac{1}{\sum_{\text{pixels}}{\left(b_{j}^{\text{ref}}\right)}^{2}}\sum_{\text{pixels}}{\left(b_{j}^{n}-b_{j}^{\text{ref}}\right)}^{2}.$$

Here $b_{j}^{n}$ is the brightfield pixel intensity at time $t_{n}$ in the jth pixel, whereas $b_{j}^{\text{ref}}$ is the brightfield pixel intensity in the jth pixel at a reference time point when the cells are at rest (not contracted). A typical displacement curve computed in this manner is displayed in the upper right panel of Fig. 2. The displacement curve is unitless. This simple assessment of the displacement is similar to the method used in Sala et al. (2018); see Discussion below.

#### Computing displacement amplitude from displacement trace

The DA is computed by subtracting the minimum value of D from the maximum value of D over time (see upper right panel of Fig. 2). The DA is unitless, and we normalize it such that for each chip, DA = 1 for the baseline case.

### From biomarkers to biophysics

From the biomarkers described in the previous section, we may identify biophysical properties of the hiPSC‐CM cell collection by applying mathematical modelling. In this section, this will be described in more detail. The software implementing the model is available online.

### Single‐cell model: Electrophysiology

As a basis for the mathematical modelling, we use a modified version of the single‐cell hiPSC‐CM model from Jæger et al. (2020), illustrated in the left panel of Fig. 3. In this model, changes in the transmembrane potential, v (in millivolt), are governed by a set of membrane currents (in µA/µF):

(2) $$\begin{array}{cc} \frac{dv}{dt} & =-I_{\mathrm{ion}}, \end{array}$$

(3) $$\begin{array}{cc} I_{\mathrm{ion}} & =I_{\mathrm{Na}}+I_{\mathrm{NaL}}+I_{\mathrm{CaL}}+I_{\mathrm{to}}+I_{\mathrm{Kr}}+I_{\mathrm{Ks}}+I_{K1}\\ & \;+I_{\mathrm{NaCa}}+I_{\mathrm{NaK}}+I_{\mathrm{pCa}}+I_{\mathrm{bCl}}+I_{\mathrm{bCa}}+I_{f}. \end{array}$$

The single‐cell model includes representation of dynamic intracellular calcium concentrations in five intracellular compartments, as illustrated in Fig. 3. The difference between the formulation used in this study and the formulation in Jæger et al. (2020) is that we also include a dynamic intracellular sodium concentration, incorporated like in Jæger, Wall et al. (2021).

### Single‐cell model: Mechanical contraction

In the single‐cell model, we have also included a model for cell contraction, based on the model from Rice et al. (2008). In our model, the cell length, $l_{x}$, is governed by an active contraction force, $F_{a}$, a passive force acting to restore the cell to its resting length, $F_{p}$, and a viscosity force, $F_{v}$, representing the cell's resistance to deformation. The mechanical contraction of the cell is assumed to be governed by Newton's second law. More specifically, the cell length, l, is modelled by

(4) $$\begin{array}{cc} m\frac{d^{2}l}{dt^{2}} & =-(F_{a}+F_{p}+F_{v}), \end{array}$$

where m is the mass of the cell. The specific formulations used for each of these forces are specified below.

To rewrite eqn (4) to a system of ordinary differential equations (ODEs), we first note that $F_{v}=k_{v}\frac{d(l-l_{0})}{dt}$ (see below), where $l_{0}$ is the resting length of the cell. Inserting this into eqn (4) yields

(5) $$\begin{array}{cc} m\frac{d^{2}l}{dt^{2}}+k_{v}\frac{d(l-l_{0})}{dt} & =-(F_{a}+F_{p}). \end{array}$$

This can be rewritten as

(6) $$\begin{array}{cc} \frac{d}{dt}\left(m\frac{dl}{dt}+k_{v}\left(l-l_{0}\right)\right) & =-(F_{a}+F_{p}). \end{array}$$

Introducing the variable $L=m\frac{dl}{dt}+k_{v}\left(l-l_{0}\right)$, eqn (6) can be expressed as

(7) $$\begin{array}{cc} \frac{dL}{dt} & =-(F_{a}+F_{p}), \end{array}$$

(8) $$\begin{array}{cc} \frac{dl}{dt} & =\frac{L-k_{v}\left(l-l_{0}\right)}{m}. \end{array}$$

The system (7)–(8) is used to model the mechanical contraction in terms of the cell length in the single‐cell model.

*Active force*, $F_{a}$ The active contraction force is activated by an increased cytosolic calcium concentration which cause calcium to bind to troponin. The subsequent myosin–actin cross‐bridge dynamics are represented using a Markov model, illustrated in the right panel of Fig. 3. The force is expressed as

(9) $$F_{a}={\bar{F}}_{a}P,$$

where ${\bar{F}}_{a}$ is a model parameter representing the maximal active force and P is the fraction of the cells' potential cross‐bridges that have undergone the power stroke, represented by the relative occupancy in state P in the cross‐bridge Markov model (right panel of Fig. 3). The Markov model consists of the following four states:

R The non‐permissive (resting) state: In this state, the myosin‐binding site on the actin is not exposed.
D The permissive detached state: In this state, the myosin‐binding site on the actin is exposed, but myosin is not bound (or only weakly bound) to the binding site.
X The cross‐bridge prepower stroke state: In this state, myosin is strongly bound to actin, and the power stroke has not happened yet.
P The cross‐bridge post power stroke state: In this state, myosin is strongly bound to actin, and the power stroke has occurred.

The relative occupancy of these states, denoted by R, D, X and P, is governed by

(10) $$\begin{array}{cc} \frac{dR}{dt} & =k_{DR}D-k_{RD}R, \end{array}$$

(11) $$\begin{array}{cc} \frac{dD}{dt} & =k_{RD}R+k_{PD}P+k_{XD}X-\left(k_{DR}+k_{DX}\right)D, \end{array}$$

(12) $$\begin{array}{cc} \frac{dX}{dt} & =k_{DX}D+k_{PX}P-\left(k_{XD}+k_{XP}\right)X, \end{array}$$

(13) $$\begin{array}{cc} \frac{dP}{dt} & =k_{XP}X-\left(k_{PX}+k_{PD}\right)P. \end{array}$$

Here, the transition rates between all the states are assumed to be constant, except for the transition between the R and D states, representing the movement of tropomyosin on the actin, exposing myosin‐binding sites on actin in response to calcium being bound to troponin on the thin filaments. These rates are given by

(14) $$\begin{array}{cc} k_{RD} & =k_{RD}^{0}\frac{{\bar{b}}_{t}^{h_{m}}}{K_{m}^{h_{m}}+{\bar{b}}_{t}^{h_{m}}}, \end{array}$$

(15) $$\begin{array}{cc} k_{DR} & =\min\left(\frac{1}{k_{RD}},100\right), \end{array}$$

where ${\bar{b}}_{t}$ is the relative amount of troponin with ${\mathrm{Ca}}^{2+}$ bound to it.

*Passive force*, $F_{p}$. The passive force acts to restore the cell to its resting length and is expressed in the same form as in (Rice et al., 2008). It is given by

(16) $$F_{p}=\left\{\begin{array}{cc} a_{m}\left(e^{b_{m}\left(l-l_{0}\right)}-1\right) & \text{if}\;l\geq l_{0},\\ -a_{m}\left(e^{b_{m}\left(l_{0}-l\right)}-1\right) & \text{if}\;l<l_{0}. \end{array}\right.$$

Here, $a_{m}$ and $b_{m}$ are model parameters, and $l_{0}$ is the resting length of the cell.

*Viscosity force*, $F_{v}$. The viscosity force represents the cell fluid's resistance to deformation. It is assumed to follow Newton's law of viscosity, which states that the shear stress, τ, is proportional to the velocity of deformation, that is

$$\tau =\mu \frac{d(l-l_{0})}{dt},$$

where µ is the viscosity, and $\frac{d(l-l_{0})}{dt}$ is the velocity of deformation. The shear stress, τ, can be expressed as the viscosity force divided by the cross‐sectional area parallel to the force ($\tau =\frac{F_{v}}{A}$). The viscosity force can thus expressed as

(17) $$F_{v}=k_{v}\frac{d(l-l_{0})}{dt},$$

where $k_{v}$ is the viscosity parameter defined as μ·A.

#### Cell‐based spatial model of a collection of cells: Electrophysiology

Using the model described above to represent the dynamics of a single cell, we apply a cell‐based spatial model to model the collection of hiPSC‐CMs in each MPS. The spatial model is based on the Kirchhoff's network model (KNM) (Jæger & Tveito, 2023b), which represents each cell and an associated extracellular compartment as a computational node. Such a detailed representation of each cell is warranted for the uneven distribution of cells located in the chips (see Figs 5 and 6). More specifically, we apply a version of the simplified KNM (SKNM) (Jæger & Tveito, 2023c), which has been shown to provide very similar solutions to the full KNM (Jæger & Tveito, 2023c; Jæger et al., 2024b). The SKNM can be written as

(18) $$\begin{array}{cc} \frac{dv_{i}}{dt} & =\eta_{i}\sum_{j\in N_{i}}G^{i,j}\left(v_{j}-v_{i}\right)-I_{\mathrm{ion}}^{i}\left(v_{i},s_{i}\right), \end{array}$$

(19) $$\begin{array}{cc} \frac{ds_{i}}{dt} & =S_{i}\left(v_{i},s_{i}\right),\; \end{array}$$

for each cell i, where $N_{i}$ is a collection of all the cells connected to cell i, $\eta_{i}$ is a lumped parameter (in (µF)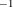) described below, $v_{i}$ is the transmembrane potential of cell i (in millivolts), $G^{i,j}$ is the intercellular conductance between cells i and j (in millisiemens), $I_{\mathrm{ion}}^{i}$ is the current density of currents through the membrane of cell i (in µA/µF) given by eqn (3) above and $s_{i}$ are additional state variables (including intracellular ion concentrations and ion channel gating variables) governed by $S_{i}$ which is given by the single‐cell model.

*The intercellular conductance*, $G^{i,j}$. The intercellular conductance, $G^{i,j}$, is given by (see, e.g., Jæger & Tveito, 2023c)

(20) $$G^{i,j}=\frac{1}{\frac{l_{i,j}}{A_{i,j}\sigma_{I}}+\frac{1}{G_{g}^{i,j}}},$$

where $l_{i,j}$ is the distance between the centres of cells i and j (in centimetres), $A_{i,j}$ is the cross‐sectional area between cells i and j (in square centimetres), $\sigma_{I}$ is the intracellular conductivity (in mS/cm) and $G_{g}^{i,j}$ is the total conductance of the gap junction channels connecting cells i and j (in millisiemens). Assuming a typical value of $\sigma_{I}=4$ mS/cm, and the values from our study of $G_{g}=1.8$ nS, cell length of 25 µm and cell width of 8 µm, the second term in the denominator of (20) is $1.8\cdot 10^{6}$ kΩ, whereas the first term is 977 kΩ for longitudinal cell connections and 100 kΩ for transverse cell connections. Because the second term seems to be considerably larger than the first term in this case, we simplify eqn (20) to

(21) $$G^{i,j}=G_{g}^{i,j},$$

in our computations, where, as specified above, $G_{g}^{i,j}$ is the conductance of the gap junctions connecting cells i and j.

*The lumped* $\eta_{i}$ *parameter*. The lumped $\eta_{i}$ parameter is defined as (see Jæger & Tveito, 2023c)

(22) $$\eta_{i}=\frac{\lambda }{C_{m}A_{m}^{i}\left(1+\lambda \right)},$$

where $C_{m}=1$ µF/cm^2^ is the specific membrane capacitance, $A_{m}^{i}$ is the membrane area of cell i (in square centimetres) and λ is a (unitless) SKNM scaling factor defined such that

(23) $$G_{E}^{i,j}\approx \lambda G^{i,j},$$

where $G_{E}^{i,j}$ and $G^{i,j}$ are the extracellular and intracellular conductances, respectively. Here, following the discussion above, $G^{i,j}=G_{g}^{i,j}$, with a typical value of 1.8 nS. Furthermore, $G_{E}^{i,j}$ is defined by (see Jæger & Tveito, 2023c)

(24) $$\begin{array}{cc} G_{E}^{i,j} & =\frac{\delta_{E}A_{i,j}\sigma_{E}}{\left(1-\delta_{E}\right)l_{i,j}}, \end{array}$$

where $\sigma_{E}$ is the extracellular conductivity, with a typical value of 20 mS/cm and $\delta_{E}$ is the extracellular volume fraction with a typical value of 0.2. For cells connected in the longitudinal direction, we get a typical value of $G_{E}^{i,j}=1280$ nS, and for cells connected in the transverse direction, we get a typical value of $G_{E}^{i,j}=12,500$ nS. Consequently, λ is relatively large (approximately in the range 700–7000), which yields a value of $\frac{\lambda }{1+\lambda }$ close to 1, and therefore,

(25) $$\eta_{i}\approx \frac{1}{C_{m}A_{m}^{i}}.$$

Moreover, the surface area of a cell shaped as a rectangular cuboid with length 25 µm and width 8 µm can be approximated to $A_{m}^{i}\approx 1000$µm^2^, which yields

(26) $$\eta_{i}\approx 1\cdot 10^{5}\mu F^{-1},$$

which is the value used for $\eta_{i}$ in our simulations.

#### Cell‐based spatial model of a collection of cells: Mechanics

In the present study, we extend the SKNM framework to include a simple representation of the mechanical contraction and displacement of cells. To this end, we assume that each cell is shaped as a 3D cuboid with rectangle form in the x–y plane as illustrated in Fig. 4 *A* and that the cells are organized in a brick‐like structure illustrated in Figs 4 *B* and 6. The length of each cell is modelled by the single‐cell model described in the ‘Single‐cell model: Mechanical contraction’ section above,

(27) $$\begin{array}{cc} \frac{dl_{x}^{i}}{dt} & =L_{i}\left(l_{x}^{i},s_{i}\right), \end{array}$$

where $L_{i}$ is the model governing the dynamics of the cell length $l_{x}^{i}$ (in micrometres). The volume of the cells, $l_{x}^{i}{\left(l_{y}^{i}\right)}^{2}$, is assumed to remain constant during contraction such that the cell width, $l_{y}^{i}$ (in micrometres), is given by

(28) $$l_{y}^{i}=l_{y,0}^{i}\sqrt{\frac{l_{x,0}^{i}}{l_{x}^{i}}},$$

where $l_{y,0}^{i}=8$ µm and $l_{x,0}^{i}=25$ µm are the default (resting) cell width and length, respectively.

Moreover, we assume that mechanical forces act between connected cells, making the cells move during contraction. All forces are assumed to act on the centre point of the cell, $\phi_{i}$.

*Forces between cells*. The cells may be connected to neighbouring cells at eight possible locations, as illustrated in Fig. 4. Forces acting between connected cells are assumed to follow Hooke's law, in the sense that the force acting between a point p on cell i and a point q on a connected cell is given by

(29) $$F_{i}^{p,q}=k_{c}\left(q-p\right)$$

(in piconewton), where $k_{c}$ is a constant (in pN/µm).

*Forces between cells and pillars*. In addition, we assume that similar forces, $F_{i,p}^{q}$ (in piconewton), act to model interaction between cells and pillars that are located in the cell collection (see Fig. 6). Figure 4 *D* illustrates a situation where a cell, i, overlaps a pillar, j. In this case, the force acting to repel the cell and pillar is assumed to be proportional to the vector defining the distance between $p_{q}^{i}$ and the purple point in Fig. 4 *D*. This distance is given by

$$-(r_{j}-∥\phi_{j}-p_{q}^{i}∥)\frac{\phi_{j}-p_{q}^{i}}{∥\phi_{j}-p_{q}^{i}∥}=\left(1-\frac{r_{j}}{∥\phi_{j}-p_{q}^{i}∥}\right)\left(\phi_{j}-p_{q}^{i}\right),$$

where the initial minus sign is used to represent that this is an overlap, that is, a ‘negative’ distance. Using Hooke's law, the force acting on the cell, i, to repel it from the pillar reads

(30) $$\begin{array}{c} F_{i,p}^{p_{q}^{i}}=k_{p}\left(1-\frac{r_{j}}{∥\phi_{j}-p_{q}^{i}∥}\right)\left(\phi_{j}-p_{q}^{i}\right), \end{array}$$

and the force acting on the pillar reads

(31) $$\begin{array}{c} F_{j,p}^{p_{q}^{i}}=-F_{i,p}^{p_{q}^{i}}=k_{p}\left(1-\frac{r_{j}}{∥\phi_{j}-p_{q}^{i}∥}\right)\left(p_{q}^{i}-\phi_{j}\right). \end{array}$$

*Viscosity force*. Finally, we consider a viscosity force, $F_{v}^{i}$, representing the cells' resistance to movement. This force (in piconewton) is given by

(32) $$\begin{array}{c} F_{v}^{i}=-k_{v}\frac{d(\phi_{i}-\phi_{i}^{0})}{dt}, \end{array}$$

where $\phi_{i}^{0}$ is the initial location of cell i and $k_{v}$ is a viscosity coefficient (in ng/ms).

*Cell location model*. Using Newton's second law, we get

(33) $$\begin{array}{cc} m_{i}\frac{d^{2}\phi_{i}}{dt^{2}} & =F_{v}^{i}+\sum_{\left(p,q\right)\in C_{i}}F_{i}^{p,q}+\sum_{q\in O_{i}}F_{i,p}^{q}, \end{array}$$

for each cell i, where $m_{i}$ is the mass of the cell (in nanograms), $C_{i}$ is a collection of all connection point pairs of cell i and $O_{i}$ is a collection of all the connection points of cell i overlapping a pillar. Using the same technique as for the single‐cell case above, this can be rewritten to the following system of first‐order ODEs:

(34) $$\begin{array}{cc} \frac{dw_{i}}{dt} & =\sum_{\left(p,q\right)\in C_{i}}F_{i}^{p,q}+\sum_{q\in O_{i}}F_{i,p}^{q}, \end{array}$$

(35) $$\begin{array}{cc} \frac{d\phi_{i}}{dt} & =\frac{1}{m_{i}}\left(w_{i}-k_{v}\left(\phi_{i}-\phi_{i}^{0}\right)\right). \end{array}$$

Similarly, the corresponding system for a pillar, i, reads,

(36) $$\begin{array}{cc} \frac{dw_{p,i}}{dt} & =\sum_{q\in O_{p,j}}F_{j,p}^{q}, \end{array}$$

(37) $$\begin{array}{cc} \frac{d\phi_{p,i}}{dt} & =\frac{1}{m_{p,i}}\left(w_{p,i}-k_{v,p}\left(\phi_{p,i}-\phi_{p,i}^{0}\right)\right), \end{array}$$

where $O_{p,j}$ is a collection of all the connection points of cells overlapping the pillar, $k_{v,p}$ is the viscosity constant for the pillars and $\phi_{p,i}^{0}$ is the initial centre point of pillar i.

*The cell mass*. In our simulations, the cells are assumed to be shaped as rectangular cuboids with resting cell length 25 µm and width 8 µm. Assuming a specific weight of 1 kg/dm^3^, this corresponds to a cell weight of 1.6 ng, which is the value used in our computations.

#### Numerical methods

To perform numerical simulations, the full SKNM formulation with mechanics is formulated as a system of ODEs that are solved using the ode15s function in MATLAB.

#### Biomarker definitions from model solutions

To use the mathematical model to identify biophysical parameters of hiPSC‐CMs, biomarkers computed from MPS images are compared to biomarkers computed from model solutions. After performing a numerical simulation of the cell‐based model described above, voltage and displacement traces are extracted in a similar manner as for the MPS images described in the ‘From images to biomarkers’ section above. More specifically, for a given time step (generally set to 10 ms), an image of the cell collection is created where each cell has a colour corresponding to its transmembrane potential value ($v_{i}$) and a location matching its current location ($\phi_{i}$) and dimensions matching the current cell dimensions ($l_{x}^{i}$ and $l_{y}^{i}$). These images are set up with a resolution of about 1 µm^2^ per pixel. The voltage trace is generated by taking the average over the entire cell collection, or a selected area, just like for the images from MPS experiments. The BR, APD and CV are computed from this (or these) traces as explained in the ‘From images to biomarkers’ section. Similarly, a displacement curve is generated by first drawing images of all cells with locations and dimensions matching $\phi_{i}$, $l_{x}^{i}$ and $l_{y}^{i}$ using a specified colour for each cell that is kept constant during the simulation (see example video in the Supporting Information). The displacement curve is computed from these images using eqn (1), and the DA is computed and normalized to be 1 for the baseline case as described in the ‘From images to biomarkers’ section.

#### Model representation of individual cell collections

There is a relatively large variation between the hiPSC‐CM collections in different chips.

*Spatial cell distribution*. One aspect that differs between cell collections is the spatial distribution of cells. In Fig. 5, the cell distributions of the seven different chips considered in this study are displayed. The left panel shows the average pixel intensity of the voltage sensitive dye images for the baseline case of each chip. We observe that for C1, the chip appears to be relatively packed with cells, whereas for C2–C6, the hiPSC‐CMs are distributed in a more varying manner. Furthermore, circular pillars are clearly visible and located within the cell collections of, for example, C2, C4 and C7.

In the right panel of Fig. 5, we show the location of the cells in the mathematical model representation of each chip. These representations have been generated by considering the images in the left panel, and applying the following steps:

1. Rotate the image so that the longitudinal chip direction matches the direction of the x‐axis.
2. Apply moving average spatial smoothing along the x‐axis (using the MATLAB function smoothdata).
3. Determine a suitable threshold pixel intensity value and classify each pixel as either ‘with cell’ or ‘without cell’ according to whether the pixel intensity is above or below this threshold value.
4. Generate a brick structure of cells in an area with dimensions covering the cell collection. Remove all cells whose midpoints are located in a pixel classified as ‘without cell’.

In Fig. 6, the model representation of the cell collection corresponding to chip C2 is displayed in a larger view. In this figure, the pillars located within the cell collection are also displayed as brown circles. Note that in the model, we only consider a single sheet of cells. That is, we consider cells connected in the x‐ and y‐directions, but not in the z‐direction.

*Synchronous or non‐synchronous cells*. Another aspect of chip‐to‐chip variation is whether the cells in the chips beat synchronously, or whether an electrical wave is initiated in one part of the chip and subsequently travels through the cell collection. Furthermore, the speed with which this wave is moving may vary between chips. For instance, the cells in Chip C1 display a slowly moving wave (see left panel of Fig. 7), whereas the cells in Chip C4 display a fast wave (see left panel of Fig. 8). By adjusting the cell‐to‐cell conductance parameter, $G^{i,j}$, in the model to make the CV in the model match the CV in the experiment, we may identify how strong the gap junction connection between cells is in the different chips. However, because the time step used in the optical measurements is typically 10–20 ms, and the maximal length of the chips is approximately 1500 µm, it is difficult for the current measurements to capture details of travelling waves if the CV is faster than about 1500 µm/15 ms = 10 cm/s.

**Figure 8:**
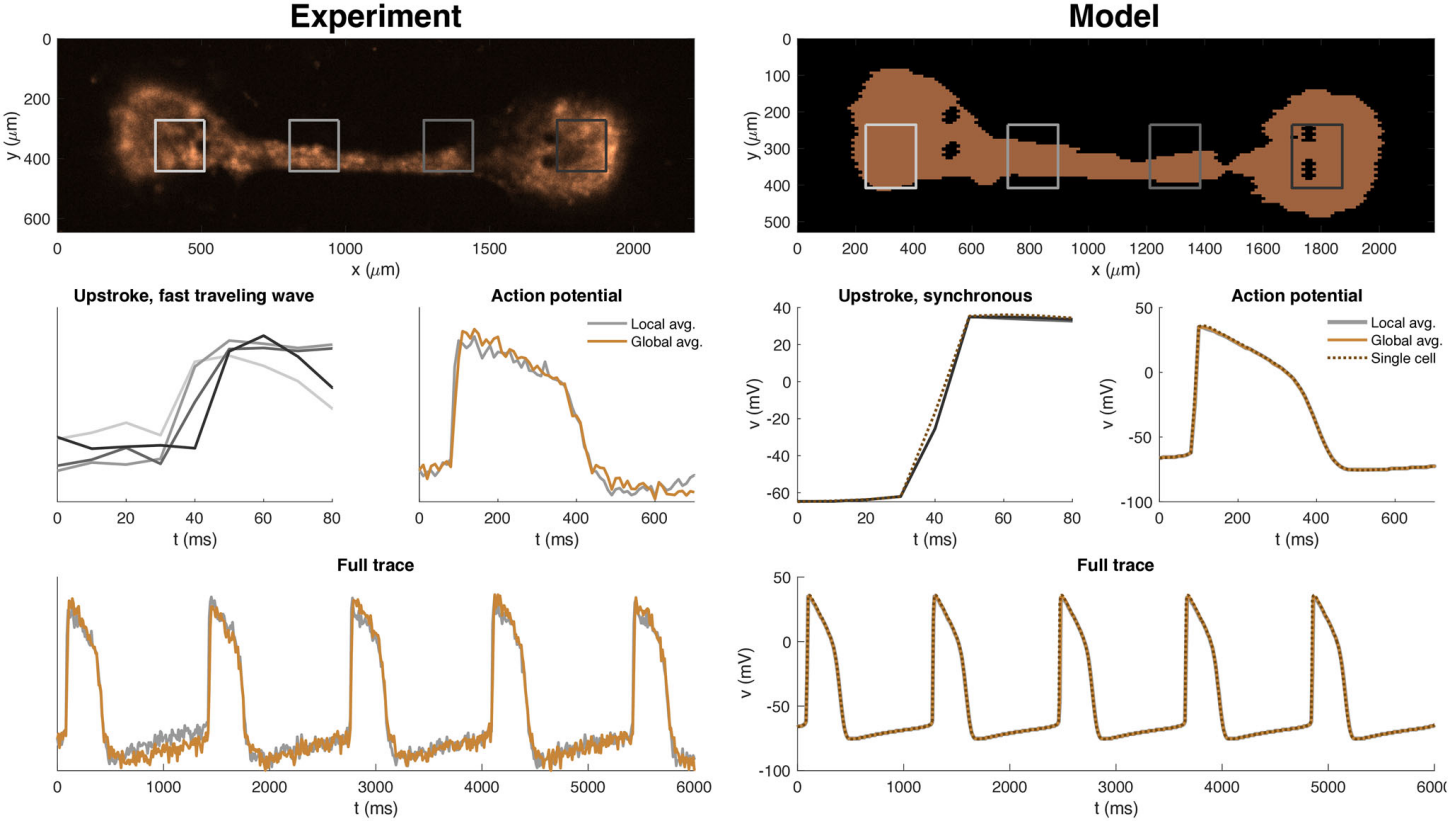
Illustration of (almost) synchronous cells in Chip C4. In the experiment (left panel), the action potential upstroke taken from four locations in the chip (see upper panel) happens almost synchronously (CV > 10 cm/s). The global average action potential or global average full voltage trace (taken over the entire chip) is almost identical to local averages (taken in the second local area). In the model, we assume that the cells beat synchronously. We compare the global average, a local average and the solution of a single‐cell simulation. All three solutions are virtually indistinguishable.

*Electrophysiological and mechanical cell properties*. In addition to the variation in travelling wave properties and the spatial distribution of cells displayed in Fig. 5, the cell collections display variations in other electrophysiological and mechanical properties. For instance, for the chips considered in this study, the baseline (no‐drug) APD varies between about 220 and 470 ms and the BR varies between 0.6 and 1.0 Hz. To represent these different properties, parameters of the single‐cell model are adjusted for each cell collection. The baseline parameter values used for each chip are provided in the Supporting Information.

### Identification of drug effects

In this study, we use the mathematical model described above to identify drug effects in terms of changed biophysical parameters. More specifically, our approach amounts to adjusting the parameters of the mathematical model so that the biomarkers computed from the model solution match as well as possible the biomarkers computed from the MPS images of hiPSC‐CMs under the influence of a drug. We perform these parameter adjustments by handtuning. We consider the six drugs listed in Table 2 with two doses of each drug. The effect of these drugs is known from literature, and we adjust the biophysical parameters that are known to be changed by the drug.

**Table 2:** List of considered drugs with mechanism of action and affected biophysical parameters.

		**Affected**
**Drug**	**Mechanism of action**	**biophysical**
		**parameters**
Flecainide	Multichannel blocker	$g_{\mathrm{Kr}}$, $g_{\mathrm{Na}}$, $g_{\mathrm{CaL}}$
Quinidine	Multichannel blocker	$g_{\mathrm{Kr}}$, $g_{\mathrm{Na}}$, $g_{\mathrm{CaL}}$
Nifedipine	L‐type calcium channel blocker	$g_{\mathrm{CaL}}$
Verapamil	L‐type calcium channel and hERG blocker	$g_{\mathrm{Kr}}$, $g_{\mathrm{CaL}}$
Blebbistatin	Myosin blocker	$k_{RD}$
Omecamtiv	Myosin activator	$k_{DX}$, $k_{XD}$, $k_{PD}$

The drugs flecainide and quinidine are known to affect several ion channel conductances, and for the doses considered in this study, effects on the conductance of the rapidly activating (hERG) potassium current, $g_{\mathrm{Kr}}$, the fast sodium current, $g_{\mathrm{Na}}$, and the L‐type calcium current, $g_{\mathrm{CaL}}$, are relevant (Crumb et al., 2016; Kramer et al., 2013). Nifedipine is an L‐type calcium channel blocker (Gibson et al., 2014; Kramer et al., 2013), and we adjust the parameter $g_{\mathrm{CaL}}$. Similarly, verapamil is a blocker of the L‐type calcium channels and the rapidly activating (hERG) potassium channels, and we adjust the parameters $g_{\mathrm{Kr}}$ and $g_{\mathrm{CaL}}$ (Crumb et al., 2016; Kramer et al., 2013).

The drugs blebbistatin and omecamtiv are known to affect the mechanical properties of CMs. In particular, blebbistatin is a myosin blocker that has been shown to block myosin heads in a state where actin cannot bind to them (Kovács et al., 2004). In the mathematical model, we let this be represented by a reduction in the transition rate $k_{RD}$ in the myosin–actin cross‐bridge cycle Markov model displayed in the right panel of Fig. 3. This is the rate of transition from the state R, where the myosin‐binding site on the actin is not exposed, to the state D, where the myosin‐binding site on actin is exposed. Omecamtiv, on the contrary, is known as a myosin activator. It has been shown to accelerate the cross‐bridge cycle step from the weakly bound to the strongly bound state and increases the time myosin is bound to actin (Planelles‐Herrero et al., 2017; Teerlink, 2009). In the mathematical model, we let this be represented by an increase in $k_{DX}$ to represent the acceleration from the weakly bound state to the strongly bound state, as well as a reduction in the transition rates $k_{XD}$ and $k_{PD}$ to increase the time when myosin is bound to actin.

### Use of artificial intelligence‐generated content

During the preparation of this manuscript, the authors utilized the ChatGPT4 language model to enhance the language quality for contributions from non‐native English speakers. Subsequent to this automated assistance, the authors rigorously reviewed and edited the manuscript to ensure its accuracy and integrity. The authors assume full responsibility for the content of the publication.

### Ethics

There was no ethics approval required for this study. Our general use of stem cell‐derived cardiomyocytes was assessed by the UC Berkeley Stem Cell Oversight Committee (SCRO), who determined that only notification of the use was required for the specific application. This notification was accepted with a SCRO reference number 2020‐0‐042.

## Results

In this section, we demonstrate how data from MPSs can be interpreted to determine the values of biophysical parameters characterizing hiPSC‐CMs by using mathematical modelling. First, we compare properties of spatial and single‐cell modelling and investigate how biomarker values are affected by biophysical parameter changes in the model. Next, we show results of adjusting parameter values in the model to match MPS measurements of hiPSC‐CMs exposed to drugs and compare the identified biophysical parameter changes to effects reported in literature.

### Spatial or single‐cell modelling

In this section, we will investigate differences between spatial or single‐cell modelling and investigate what type of modelling is required in different situations.

#### Spatial modelling is required to represent non‐synchronous cell collections

A slow excitation wave travelling through Chip C1 is displayed in the left panels of Fig. 7. The upper panel illustrates seven areas where local average voltage traces are recorded. The voltage trace for each local area is illustrated in the lower left panel. Using the approach described in the ‘From images to biomarkers’ section above, the CV is estimated to be 1.4 cm/s. In the lower right panel, we compare the average trace in one of the local areas to the global average trace over all the seven areas. We observe that the action potential shape differs considerably between the local and global averages, illustrating the need for taking local averages in the case of non‐synchronous cells with slow excitation waves. In the right panels of Fig. 7, we report results of a model simulation using biophysical parameters fitted to the experimental biomarkers. For instance, the CV is fitted to be 1.4 cm/s and the cell‐to‐cell conductance, $G^{i,j}$, is found to be 1.7 nS. In the simulation, the travelling wave is for simplicity initiated by applying a stimulation current to the leftmost cells. A video showing the fluorescence images from the experiment and the model solution is found in the Supporting Information.

#### Single‐cell modelling is sufficient to capture electrophysiological properties of synchronous and almost synchronous cells

In the left panels of Fig. 8, we show a similar example for Chip C4, where the travelling wave moves much faster, with a CV > 10 cm/s. The action potential upstroke for each of the four local areas illustrated in the upper panel is displayed in the middle left panel. The next panels show the action potential and the full voltage trace for this experiment, and we compare the average trace computed from one of the local areas to the global average over the entire chip. We observe that the action potential shape and the BR is virtually identical for the global and local averages and therefore conclude that the global average is sufficient in such cases. In addition, we conclude that it is adequate to let the cells beat in synchrony in mathematical model representations of such cell collections. The model solution associated with Chip C4 is displayed in the right panels of Fig. 8. In this case, we also consider the solution of the single‐cell model described in the ‘Single‐cell model: Electrophysiology’ section above. We observe that for synchronously beating cells, the local and global averages of spatially resolved simulations are practically identical to the single‐cell model. For the electrophysiological properties of the cells, a single‐cell model could, therefore, be sufficient to determine relevant biophysical parameters of synchronously beating cells.

#### Spatial modelling may be required to correctly capture mechanical perturbations

In Fig. 9, we consider the case of a drug applied to the cells in Chip C4. More specifically, we consider the application of 0.1 µM of the L‐type calcium channel blocker nifedipine. The voltage and displacement traces from this experiment are displayed in the leftmost panel of Fig. 9. We observe that the APD is shortened and that the DA is reduced by the application of the drug. In the centre panel, we show the results of a spatial simulation with the effect of the drug represented by a 50% reduction in the conductance of the L‐type calcium current, $g_{\mathrm{CaL}}$. We observe that the APD and DA reductions appear to be represented well in the spatial model simulations.

**Figure 9:**
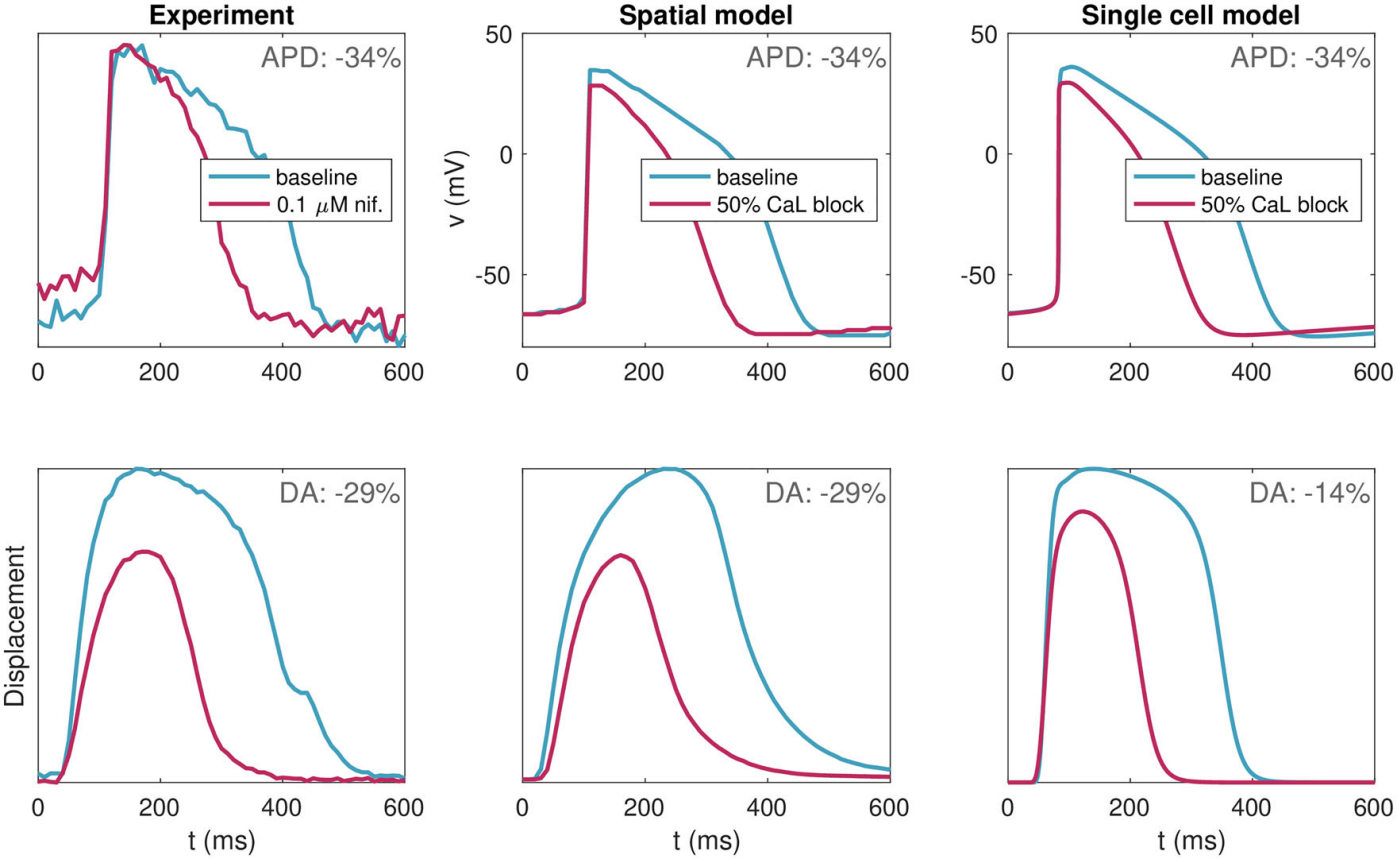
Measured and simulated voltage and displacement traces for the cells in Chip C4 in the baseline case and after the application of 0.1 µM of the L‐type calcium channel blocker, nifedipine. In the simulations, the effect of the drug is represented by reducing $g_{\mathrm{CaL}}$ by 50%. Furthermore, in the single‐cell simulation, the displacement is defined as the shortening of the cell length, whereas for the experiment and the spatial simulation, the displacement is defined as described in the ‘Computing displacement traces from brightfield images’ section.

In the rightmost panel of Fig. 9, we have performed a similar simulation using a single‐cell model. The effect of the drug is still represented by a 50% reduction in $g_{\mathrm{CaL}}$. In this case, we let the displacement be represented by the single‐cell length shortening present in the single‐cell simulation. We observe that the transmembrane potential computed in the single‐cell model appears to be very similar to that computed from the spatial simulation and the experiment data. Because the cells in Chip C4 are almost synchronous, this is consistent with the observation from Fig. 8 that a single‐cell model is sufficient to represent the electrophysiological properties of synchronous cells. For the displacement, however, we observe that the reduction in DA computed from the cell length shortening in the single‐cell model differs a bit from that observed in the experimental measurements and in the spatial simulation. In the experiment and the spatial simulation, DA is reduced by 29%, whereas in the simulation of the single‐cell model, DA is only reduced by 14%. This indicates that spatial modelling might be required to properly capture changes in the DA.

### Sensitivity of biomarkers in response to biophysical parameter changes

To investigate the feasibility of using the biomarkers defined in the ‘From images to biomarkers’ section to identify changes in biophysical parameters, we investigate the biomarkers' sensitivity to parameter changes by performing simulations of the model fitted to Chip C1 (see Fig. 7). We consider a synchronous case with uniformly spontaneously beating cells, and a non‐synchronous case, where excitation waves are initiated in the left part of the cell collection. To be able to measure the BR in both cases, the excitation waves are in this case initiated by a constant 10% reduction in the conductance of the inward rectifier potassium current, $g_{K1}$, in the leftmost cells, making these cells more easily excited.

Figure 10 reports the changes in biomarker values (in percentage) resulting from a 25% reduction in each of the main biophysical parameters considered in this study. We observe that reducing $g_{\mathrm{Kr}}$ primarily leads to an increased APD and that reducing $k_{RD}$ or $k_{DX}$ leads to a reduced DA. Oppositely, reducing $k_{XD}$ or $k_{PD}$ leads to an increased DA. A reduction in the cell‐to‐cell conductance, $G^{i,j}$, leads to a reduction in the CV in the non‐synchronous case, but to no biomarker changes in the synchronous case. This demonstrates that non‐synchronous data are required to infer information about the cell‐to‐cell conductance. Reductions in $g_{\mathrm{Na}}$ and $g_{\mathrm{CaL}}$ both affect all the considered biomarkers, but reducing $g_{\mathrm{Na}}$ primarily reduces the BR and the CV. Reducing $g_{\mathrm{CaL}}$ results in reduced APD and DA and increased BR and CV.

**Figure 10:**
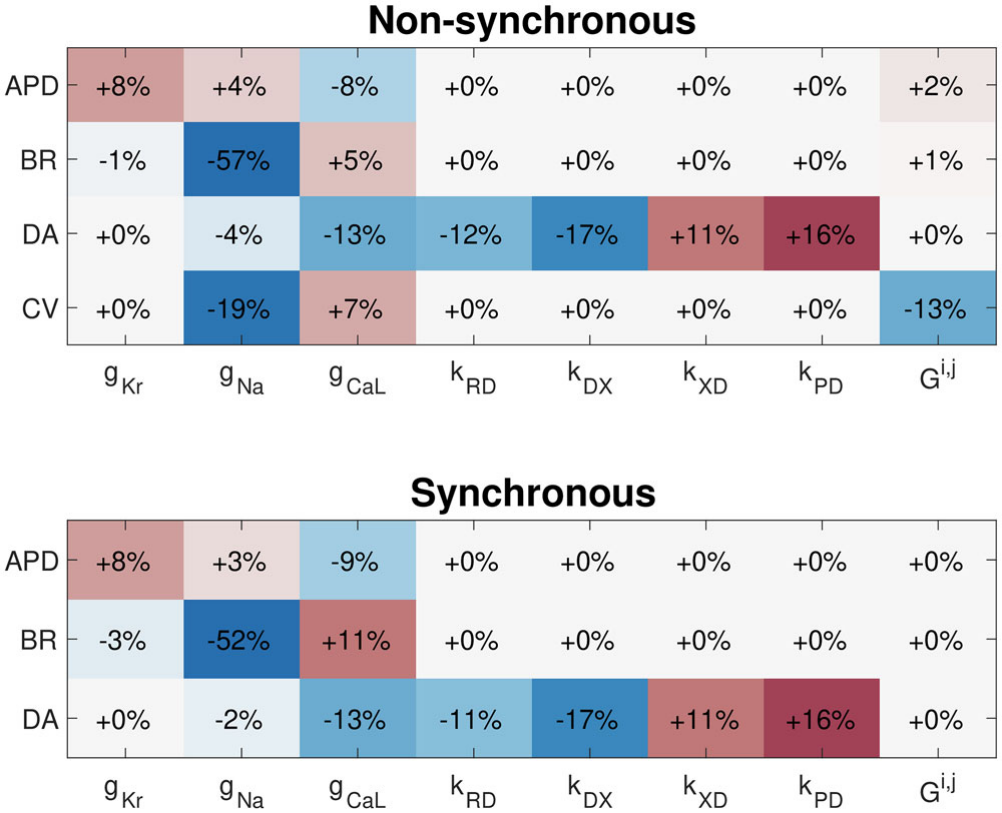
Sensitivity of the biomarkers to changes in the biophysical parameters $g_{\mathrm{Kr}}$, $g_{\mathrm{Na}}$, $g_{\mathrm{CaL}}$, $k_{RD}$, $k_{DX}$, $k_{XD}$, $k_{PD}$ and $G^{i,j}$. The figures display the change of biomarker value (in percentage) resulting from a 25% reduction in the parameter values. The biomarkers are computed from model simulations using the parameterization and spatial cell distribution of Chip C1 (see Fig. 7). In the upper panel, we consider non‐synchronous cells with an excitation wave initiated by a 10% weaker $g_{K1}$ in the leftmost cells. In the lower panel, we consider synchronously beating cells with uniform cell properties.

### Identification of drug effects

As seen in Fig. 10, changes in the biophysical parameters affected by the six drugs listed in Table 2 all result in detectable changes in the biomarkers described in the ‘From images to biomarkers’ section. In this section, we show results of adjusting the biophysical parameters in the model to make the biomarkers computed from the model solutions match as well as possible the biomarkers computed from MPS images of hiPSC‐CMs in the presence of drugs. For each of the drugs listed in Table 2, we will consider the baseline (no‐drug) case and two different drug doses.

#### Flecainide

We first consider an experiment in Chip C2 where 1 and 10 µM of flecainide are applied to the hiPSC‐CMs. In the left panels of Fig. 11, we study the excitation waves moving through the cells in the baseline, 1 and 10 µM cases. In the baseline case, the cells in the four considered areas of the chip (see upper panel) all seem to be excited in almost complete synchrony, whereas in the cases with 1 and 10 µM of flecainide, the cells are not completely synchronous. In the 1 µM case, an excitation wave moves quite fast through the cell collection with a CV above 10 cm/s. In the 10 µM case, the wave moves more slowly with a CV estimated to 5.9 cm/s.

**Figure 11:**
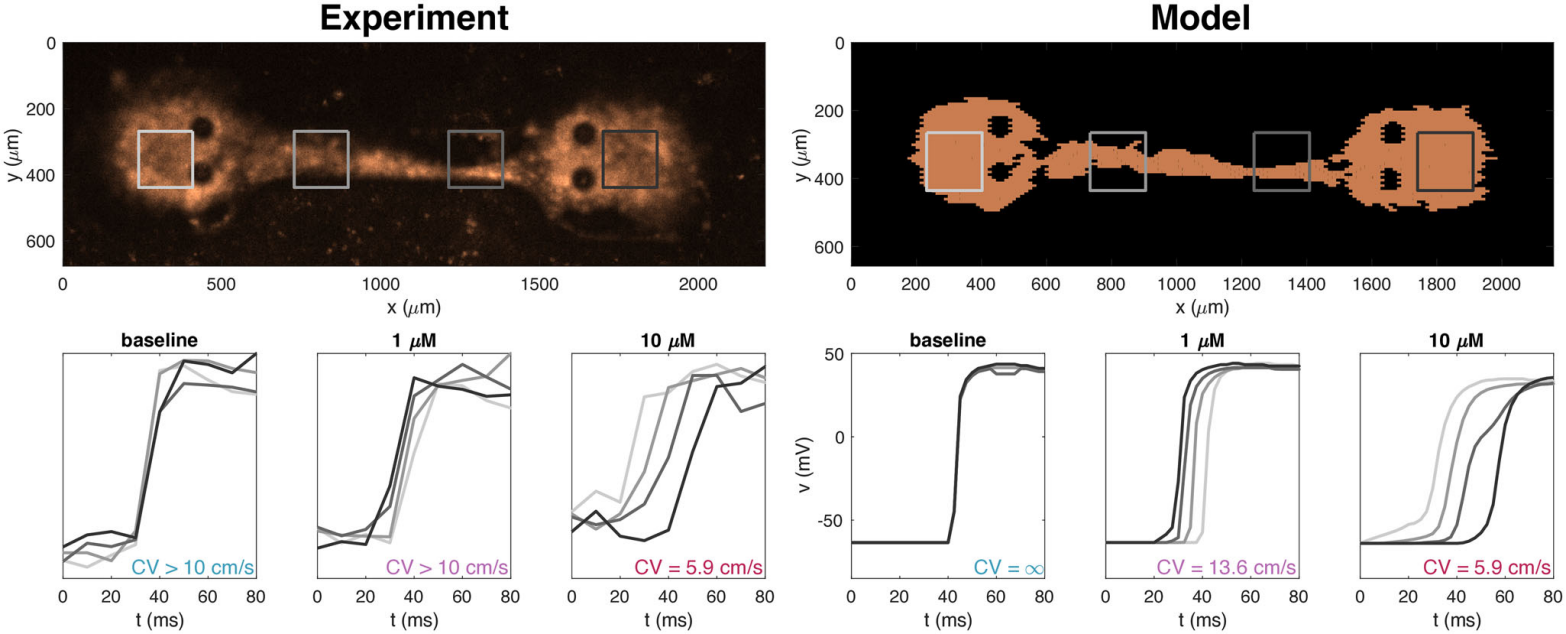
Flecainide. Average action potential upstroke in four local areas of Chip C2 in the baseline case and after application of 1 and 10 µM of flecainide. The upper panel illustrates the local areas used to define the action potential upstrokes in the lower panel. The left panels show optical measurements from an experiment, and the right panels show the model solution. The effect of the drug is modelled as described in Table 9.

Interestingly, the waves in the 1 and 10 µM cases appear to be initiated in opposite parts of the cell collection (wave from right to left for 1 µM and from left to right for 10 µM). To represent this in the model simulations, we simply apply a stimulation current to the rightmost or leftmost cells in these two cases with a frequency matching the measured BR from the experiment. In the control case, all cells are similarly stimulated with frequency given from the measurements.

The right panels of Figs 11 and 12 show the excitation waves in the model solutions. Here, the CV is 13.6 cm/s in the 1 µM case and 5.9 cm/s in the 10 µM case. This was achieved for a cell‐to‐cell conductance of $G^{i,j}$ = 180 nS and a sodium current conductance, $g_{\mathrm{Na}}$, reduction of 10% for 1 µM of flecainide and of 80% for 10 µM of flecainide. This is in rough agreement with properties of sodium current block by flecainide from literature, with blocking percentages in the range 3%–19% for 1 µM and in the range 62%–69% for 10 µM (Crumb et al., 2016; Kramer et al., 2013; Qu & Vargas, 2015).

**Figure 12:**
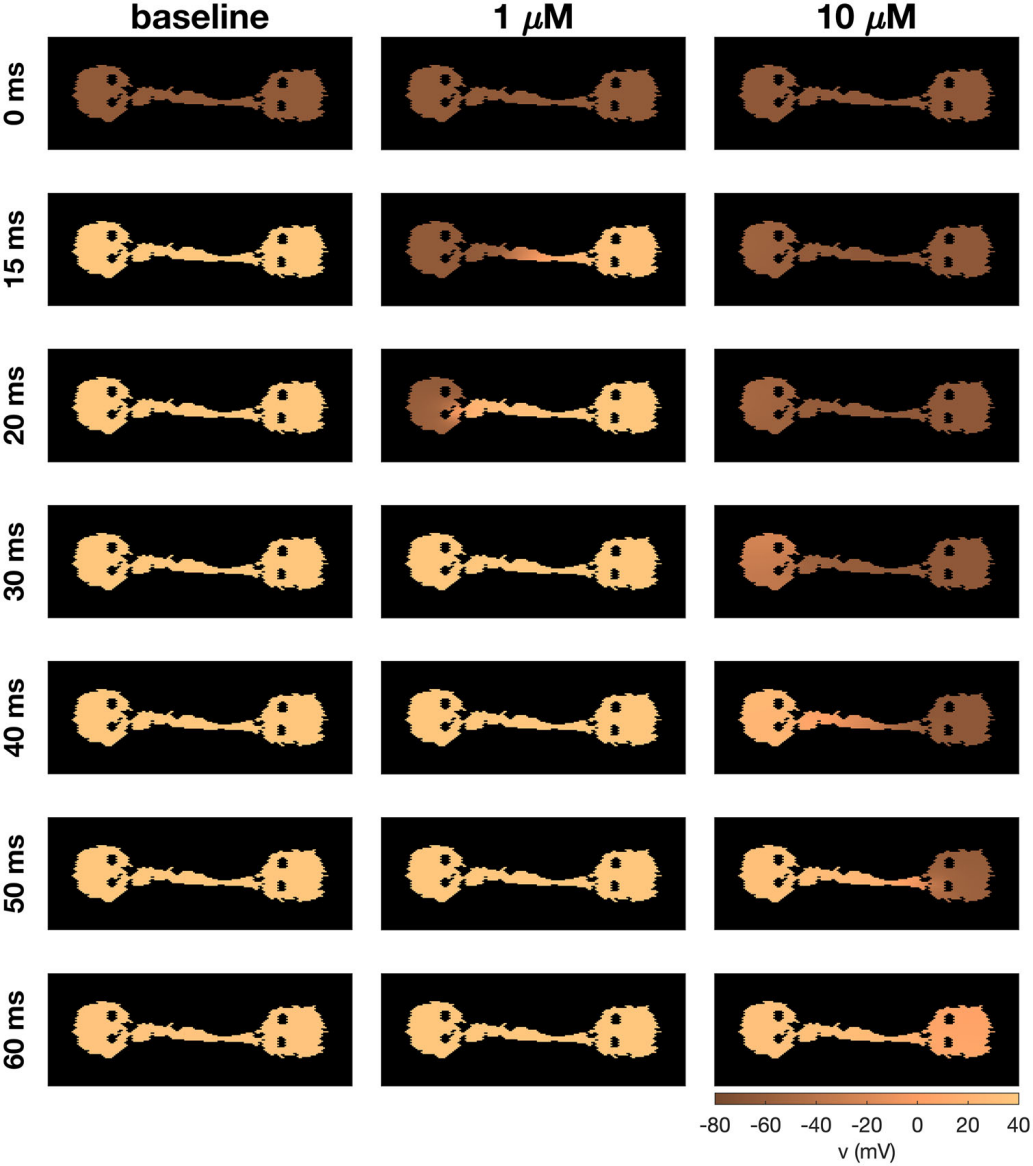
Flecainide. Snapshots of the model solutions showing electrical waves traversing Chip C2 after the application of 1 and 10 µM of flecainide. In the baseline case, no waves are present because the cells in the chip beat synchronously.

The left panels of Fig. 13 show the full voltage traces, the action potential and the displacement curves recorded in a local area in the centre of Chip C2 in the baseline case and for 1 and 10 µM of flecainide. We observe that the drug appears to reduce the BR, increase the APD and reduce the DA in a dose‐dependent manner. This is confirmed in the left panel of Table 3, which reports the values of each of the considered biomarkers. In the right panels of Fig. 13 and Table 3, the corresponding curves and biomarkers for the fitted model solution are displayed. We observe that the model solution replicates the APD prolongation observed in the experiments quite well. In addition, the DA increases slightly for 1 µM and is considerably reduced for 10 µM both in the experiment and in the model. The BR in the model also matches the experiment, but in this case, this similarity is enforced by an applied stimulation current as explained above.

**Table 3:** Flecainide

	Experiment			Model		
Biomarker	Baseline	1 µM	10 µM	Baseline	1 µM	10 µM
APD (ms)	370	490 (+32%)	502 (+36%)	369	491 (+33%)	503 (+36%)
BR (Hz)	0.67	0.61 (−9%)	0.53 (−21%)	0.67*	0.61* (−9%)	0.53* (−21%)
DA	1.00	1.05 (+5%)	0.54 (−46%)	1.00	1.05 (+5%)	0.58 (−42%)
CV (cm/s)	>10	>10	5.9	∞	13.6	5.9

Biomarkers computed from images recorded from experiments (left) and from the mathematical model solution (right) for the cells in Chip C2 in response to the drug flecainide. Numbers in parentheses indicate change from baseline. The effect of the drug is modelled as described in Table 9. *Enforced by stimulation.

**Figure 13:**
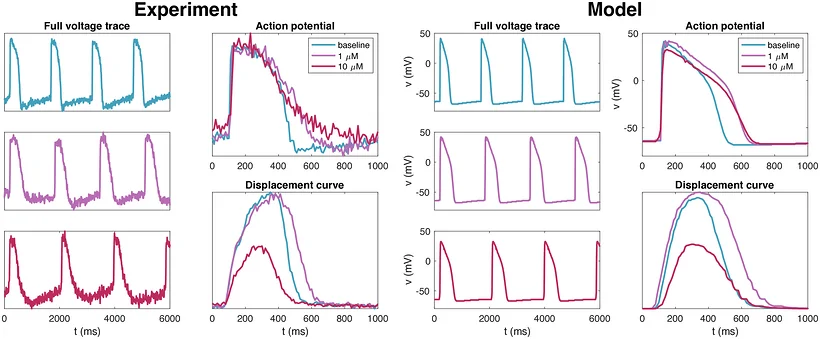
Flecainide. Full voltage traces, action potentials and displacement curves for a local area of Chip C2 in the baseline case and after application of 1 and 10 µM of flecainide. The left panels show optical measurements from an experiment, and the right panels show the model solution. The corresponding biomarker values are provided in Table 3. The effect of the drug is modelled as described in Table 9.

In addition to the $g_{\mathrm{Na}}$ reduction described above, the effect of flecainide is in the model represented by a reduction in the conductance of the rapidly activating (hERG) potassium current, $g_{\mathrm{Kr}}$, of 50% for 1 µM and 86% for 10 µM, as well as a reduction of the conductance of the L‐type calcium current, $g_{\mathrm{CaL}}$, of 1% for 1 µM and 45% for 10 µM. These blocking percentages are in relatively good agreement with blocking percentages reported in literature (see Table 9), although the reduction of $g_{\mathrm{CaL}}$ for 10 µM is somewhat stronger than the 21% reduction reported in literature (Crumb et al., 2016; Kramer et al., 2013).

**Table 4:** Quinidine

	Experiment			Model		
Biomarker	Baseline	0.1 µM	1 µM	Baseline	0.1 µM	1 µM
APD (ms)	358	413 (+15%)	545 (+52%)	356	417 (+17%)	543 (+52%)
BR (Hz)	0.78	0.77 (−1%)	0.79 (+1%)	0.77	0.79 (+3%)	0.79 (+3%)
DA	1.00	0.95 (−5%)	0.84 (−16%)	1.00	0.96 (−4%)	0.83 (−17%)

Biomarkers computed from images recorded from experiments (left) and from the mathematical model solution (right) for the cells in Chip C3 in response to the drug quinidine. Numbers in parentheses indicate change from baseline. The effect of the drug is modelled as described in Table 9.

**Table 5:** Nifedipine

	Experiment			Model		
Biomarker	Baseline	0.1 µM	1 µM	Baseline	0.1 µM	1 µM
APD (ms)	338	222 (−35%)	105 (−69%)	336	217 (−35%)	108 (−68%)
BR (Hz)	0.84	1.19 (+42%)	1.80 (+114%)	0.84	1.04 (+24%)	1.96 (+133%)
DA	1.00	0.71 (−29%)	0.03 (−97%)	1.00	0.71 (−29%)	0.00 (−100%)

Biomarkers computed from images recorded from experiments (left) and from the mathematical model solution (right) for the cells in Chip C4 in response to the drug nifedipine. Numbers in parentheses indicate change from baseline. The effect of the drug is modelled as described in Table 9

**Table 6:** Verapamil

	Experiment			Model		
Biomarker	Baseline	0.1 µM	1 µM	Baseline	0.1 µM	1 µM
APD (ms)	416	241 (−42%)	170 (−50%)	418	237 (−43%)	181 (−57%)
BR (Hz)	0.60	1.04 (+73%)	1.57 (+162%)	0.61	0.94 (+54%)	1.82 (+198%)
DA	1.00	0.41 (−59%)	0.02 (−98%)	1.00	0.39 (−61%)	0.11 (−89%)

Biomarkers computed from images recorded from experiments (left) and from the mathematical model solution (right) for the cells in Chip C5 in response to the drug verapamil. Numbers in parentheses indicate change from baseline. The effect of the drug is modelled as described in Table 9.

**Table 7:** Blebbistatin

	Experiment			Model		
Biomarker	Baseline	0.1 µM	1 µM	Baseline	0.1 µM	1 µM
APD (ms)	393	392 (−0%)	378 (−4%)	389	389 (−0%)	389 (−0%)
BR (Hz)	0.65	0.71 (+10%)	0.71 (+10%)	0.67	0.67 (+0%)	0.67 (+0%)
DA	1.00	0.32 (−68%)	0.01 (−99%)	1.00	0.29 (−71%)	0.00 (−100%)

Biomarkers computed from images recorded from experiments (left) and from the mathematical model solution (right) for the cells in Chip C6 in response to the drug blebbistatin. Numbers in parentheses indicate change from baseline. The effect of the drug is modelled as described in Table 9.

**Table 8:** Omecamtiv

	Experiment			Model		
Biomarker	Baseline	0.1 µM	1 µM	Baseline	0.1 µM	1 µM
APD (ms)	471	497 (+6%)	517 (+10%)	472	472 (+0%)	472 (+0%)
BR (Hz)	1.00	1.00 (+0%)	1.00 (+0%)	1.00	1.00 (+0%)	1.00 (+0%)
DA	1.00	1.11 (+11%)	1.26 (+26%)	1.00	1.13 (+13%)	1.27 (+27%)

Biomarkers computed from images recorded from experiments (left) and from the mathematical model solution (right) for the cells in Chip C7 in response to the drug omecamtiv. Numbers in parentheses indicate change from baseline. The effect of the drug is modelled as described in Table 9.

**Table 9:** Parameter values' reduction and increase (in percentage) in response to the considered doses of the considered drugs

		Percentage reduction						Percentage increase
		$g_{\mathrm{Kr}}$	$g_{\mathrm{Na}}$	$g_{\mathrm{CaL}}$	$k_{RD}$	$k_{XD}$	$k_{PD}$	$k_{DX}$
Flecainide (1 µM)	Model	50	10	1				
	Literature	39–57	3–19	1–3				
	References	(Crumb et al., 2016)	(Crumb et al., 2016)	(Crumb et al., 2016)				
		(Kramer et al., 2013)	(Kramer et al., 2013)	(Kramer et al., 2013)				
		(Qu & Vargas, 2015)	(Qu & Vargas, 2015)					
Flecainide (10 µM)	Model	86	80	45				
	Literature	80–90	62–69	21				
	References	(Crumb et al., 2016)	(Crumb et al., 2016)	(Crumb et al., 2016)				
		(Kramer et al., 2013)	(Kramer et al., 2013)	(Kramer et al., 2013)				
		(Qu & Vargas, 2015)	(Qu & Vargas, 2015)					
Quinidine (0.1 µM)	Model	32		10				
	Literature	7–25	0–1	0–3				
	References	(Crumb et al., 2016)	(Kramer et al., 2013)	(Kramer et al., 2013)				
,		(Kramer et al., 2013)	(Romero et al., 2018)	(Romero et al., 2018)				
		(Romero et al., 2018)	(Mirams et al., 2011)	(Mirams et al., 2011)				
		(Po et al., 1999)						
Quinidine (1 µM)	Model	76		25				
	Literature	44–77	4–6	6–12				
	References	(Crumb et al., 2016)	(Kramer et al., 2013)	(Kramer et al., 2013)				
,		(Kramer et al., 2013)	(Romero et al., 2018)	(Romero et al., 2018)				
		(Romero et al., 2018)	(Mirams et al., 2011)	(Mirams et al., 2011)				
		(Po et al., 1999)						
Nifedipine (0.1 µM)	Model			50				
	Literature	0	0	62–79				
	References	(Kramer et al., 2013)	(Kramer et al., 2013)	(Kramer et al., 2013)				
				(Gibson et al., 2014)				
				(Di Stilo et al., 1998)				
				(Romero et al., 2018)				
Nifedipine (1 µM)	Model			99				
	Literature	3	2.3	87–95				
	References	(Kramer et al., 2013)	(Kramer et al., 2013)	(Kramer et al., 2013)				
				(Gibson et al., 2014)				
				(Di Stilo et al., 1998)				
				(Romero et al., 2018)				
Verapamil (0.1 µM)	Model	10		67				
	Literature	14–42	0	32–50				
	References	(Crumb et al., 2016)	(Crumb et al., 2016)	(Crumb et al., 2016)				
		(Kramer et al., 2013)	(Kramer et al., 2013)	(Kramer et al., 2013)				
		(Zhang et al., 1999)	(Romero et al., 2018)	(Romero et al., 2018)				
		Orvos et al. (2019)						
Verapamil (1 µM)	Model	60		85				
	Literature	61–88	0–2	69–91				
	References	(Crumb et al., 2016)	(Crumb et al., 2016)	(Crumb et al., 2016)				
		(Kramer et al., 2013)	(Kramer et al., 2013)	(Kramer et al., 2013)				
		(Zhang et al., 1999)	(Romero et al., 2018)	(Romero et al., 2018)				
		Orvos et al. (2019)						
Blebbistatin (1 µM)	Model				77			
Blebbistatin (10 µM)	Model				99			
Omecamtiv (0.1 µM)	Model					7	7	7
Omecamtiv (1 µM)	Model					15	15	15

The values identified in the model are compared to those obtained from literature. Note that no literature values were found for blebbistatin or omecamtiv.

#### Quinidine

The next considered drug compound is quinidine, applied at the doses of 0.1 and 1 µM to the hiPSC‐CMs in Chip C3. The left panels of Fig. 14 show the full voltage trace, the action potential and the displacement curve for the baseline case and for the two drug doses. For this chip (and the remaining four chips), no slow excitation waves were observed, so global averages over the entire chip are used. We observe that the BR appears to be relatively unaffected by the drug, but the APD is prolonged and the DA is reduced in a dose‐dependent manner. This is also observed in the left panel of Table 4, which reports the biomarker values and associated percentage changes from baseline.

**Figure 14:**
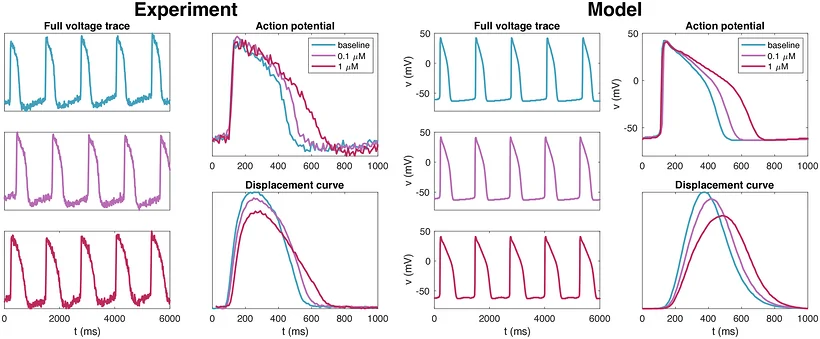
Quinidine. Full voltage traces, action potentials and displacement curves for a local area of Chip C3 in the baseline case and after application of 0.1 and 1 µM of quinidine. The left panels show optical measurements from an experiment, and the right panels show the model solution. The corresponding biomarker values are provided in Table 4. The effect of the drug is modelled as described in Table 9.

The right panels of Fig. 14 and Table 4 show the corresponding curves and biomarkers computed from the fitted model solution. We observe that the model solution replicates the APD prolongation, DA reduction and relatively unchanged BR in response to the drug doses. Note that in this case (as well as for the following drugs), no stimulation currents are applied and the BR, therefore, depends on the model parameters, as observed in Fig. 10. In the model, the drug is represented by a reduction in $g_{\mathrm{Kr}}$ of 32% for 0.1 µM and 76% for 1 µM. This is in relatively good agreement with blocking percentages from literature, in the range 7%–25% for 0.1 µM and 44%–77% for 1 µM (Crumb et al., 2016; Kramer et al., 2013; Po et al., 1999; Romero et al., 2018) (see Table 9). In addition, $g_{\mathrm{CaL}}$ is reduced by 10% for 0.1 µM and by 25% for 1 µM. This is in rough agreement, but slightly more prominent than blocking percentages from literature in the range 0%–3% for 0.1 µM and 6%–12% for 1 µM (Kramer et al., 2013; Mirams et al., 2011; Romero et al., 2018). In the model, we have not included a reduction in $g_{\mathrm{Na}}$ from quinidine, but in literature, a relatively modest reduction of about 4%–6% has been reported for 1 µM (Kramer et al., 2013; Mirams et al., 2011; Romero et al., 2018).

#### Nifedipine

The L‐type calcium channel blocker nifedipine is applied to the hiPSC‐CMs in Chip C4 in the doses of 0.1 and 1 µM. The left panels of Fig. 15 and Table 5 display the voltage and displacement curves for this experiment, as well as the associated biomarker values. We observe that the drug appears to increase the BR and reduce the APD and DA considerably in a dose‐dependent manner. For the 1 µM dose, the displacement of the cells appears to be completely gone.

**Figure 15:**
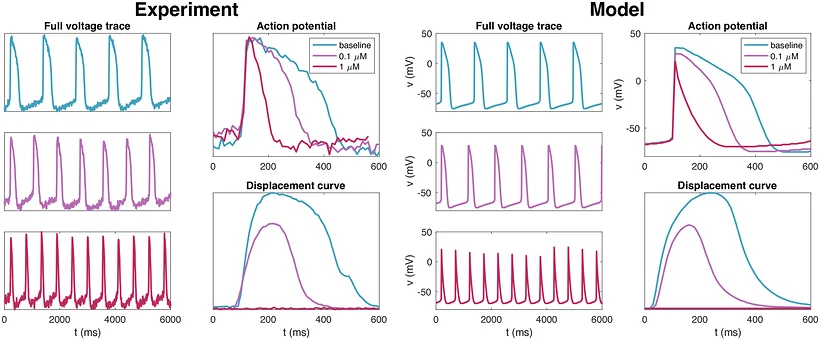
Nifedipine. Full voltage traces, action potentials and displacement curves for a local area of Chip C4 in the baseline case and after application of 0.1 and 1 µM of nifedipine. The left panels show optical measurements from an experiment, and the right panels show the model solution. The corresponding biomarker values are provided in Table 5. The effect of the drug is modelled as described in Table 9.

In the right panels of Fig. 15 and Table 5, we show the corresponding curves and biomarkers for the fitted model solution. We observe that the model accurately replicates the APD and DA reductions caused by the drug. The BR also increases by the drug in the model, but the increase is somewhat smaller than that in the experiment for the 0.1 µM dose and somewhat larger than that in the experiment for the 1 µM dose. In the model, the effect of nifedipine is represented by a reduction in $g_{\mathrm{CaL}}$ of 50% for 0.1 µM and 99% for 1 µM, quite close to the ranges of 62%–79% reduction for 0.1 µM and 87%–95% reduction for 1 µM found in literature (Di Stilo et al., 1998; Gibson et al., 2014; Kramer et al., 2013; Romero et al., 2018).

#### Verapamil

In Chip C5, we consider hiPSC‐CMs exposed to 0.1 and 1 µM of verapamil. In the left panels of Fig. 16 and Table 6, we observe that the effect of the drug on the biomarkers is quite similar to the effects of the drug nifedipine observed above. The BR is increased, and the APD and DA are reduced considerably by the drug. In the right panels of Fig. 16 and Table 6, we observe that the fitted model solution also displays a considerable BR increase and APD and DA reduction. The effect of the drug is represented by a reduction in $g_{\mathrm{CaL}}$ of 67% for 0.1 µM and 85% for 1 µM. The reduction for 1 µM is within the range of L‐type calcium channel block reported in literature (69%–91%), whereas the reduction for 0.1 µM is somewhat stronger than the range of the obtained literature values (32%–50%) (Crumb et al., 2016; Kramer et al., 2013; Romero et al., 2018). In addition, the effect of verapamil is represented by a reduction in $g_{\mathrm{Kr}}$ of 10% for 0.1 µM and of 60% for 1 µM, in quite good agreement with the ranges 14%–42% for 0.1 µM and 61%–88% for 1 µM reported in literature (Crumb et al., 2016; Kramer et al., 2013; Orvos et al., 2019; Zhang et al., 1999).

**Figure 16:**
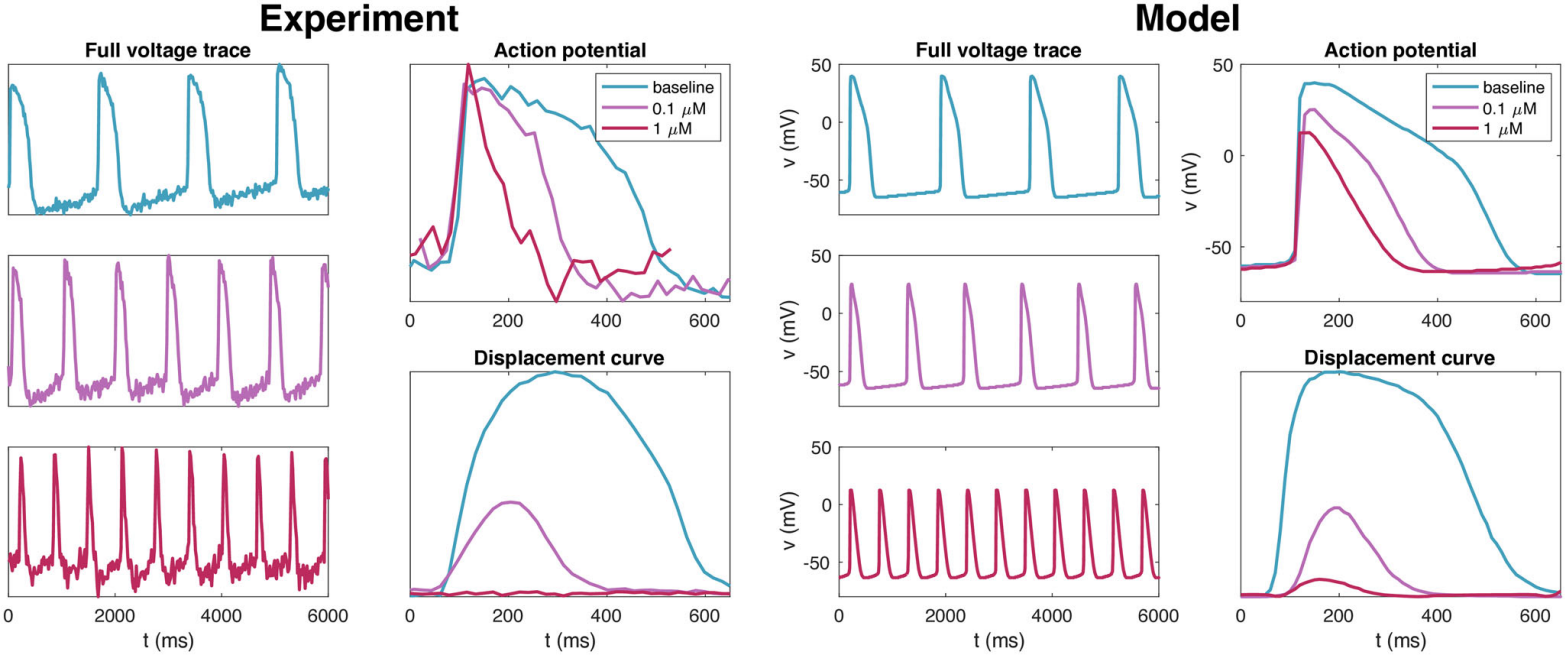
Verapamil. Full voltage traces, action potentials and displacement curves for a local area of Chip C5 in the baseline case and after application of 0.1 and 1 µM of verapamil. The left panels show optical measurements from an experiment, and the right panels show the model solution. The corresponding biomarker values are provided in Table 6. The effect of the drug is modelled as described in Table 9.

#### Blebbistatin

Next, we consider Chip C6, where the hiPSC‐CMs are exposed to 1 and 10 µM of the myosin blocker blebbistatin. In the left panels of Fig. 17 and Table 7, we observe that the drug appears to have only small effects on the APD and BR, but that the DA is considerably reduced by the drug. Indeed, for the 10 µM dose, no displacement seems to occur. In the right panels of Fig. 17 and Table 7, we observe that the fitted model solution displays no changes in APD or BR but replicates the considerable reduction in DA observed in the experiment in response to the drug. The effect of the drug is represented by reducing the transition rate $k_{DR}$ in the myosin–actin cross‐bridge Markov model from the state where the myosin‐binding site on the actin is not exposed to the state where it is exposed. The transition rate is reduced by 77% for 1 µM of blebbistatin and 99% for 10 µM of blebbistatin. We have not been able to find literature values that these reduction percentages may be compared to, but blebbistatin has been reported to cause a reduction in the myosin heads that are able to bind to actin (Kovács et al., 2004), which is quite consistent with a reduction in $k_{RD}$ in the model.

**Figure 17:**
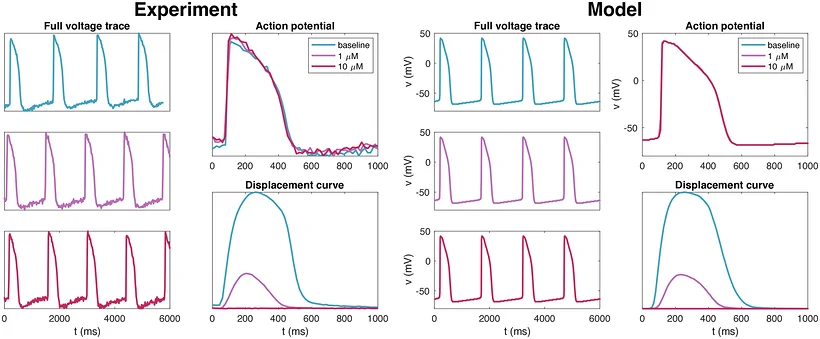
Blebbistatin. Full voltage traces, action potentials and displacement curves for a local area of Chip C6 in the baseline case and after application of 1 and 10 µM of blebbistatin. The left panels show optical measurements from an experiment, and the right panels show the model solution. The corresponding biomarker values are provided in Table 7. The effect of the drug is modelled as described in Table 9.

#### Omecamtiv

The last considered drug in this study is the myosin activator omecamtiv. This drug is applied to the hiPSC‐CMs in Chip C7 in the doses of 0.1 and 1 µM. The left panels of Fig. 18 and Table 8 reveal that the drug has no or only small effects on the BR and APD, but that the DA is increased in a dose‐dependent manner by the drug. In the right panels of Fig. 18 and Table 8, we observe that the fitted model solution also display an increase in the DA, but no drug effects on the BR or APD. The effect of the drug is represented by an increase in the transition rate $k_{DX}$ and a reduction in the transition rates $k_{XD}$ and $k_{PD}$ in the myosin–actin cross‐bridge Markov model. The transition rates are reduced or increased by 7% for 0.1 µM and by 15% for 1 µM of omecamtiv. We have not found values in literature that these reductions in transition rate can be compared to, but omecamtiv has been reported to accelerate the cross‐bridge cycle step from the weakly bound to the strongly bound state and increases the time myosin is bound to actin (Planelles‐Herrero et al., 2017; Teerlink, 2009), consistent with these transition rate adjustments (see Fig. 3).

**Figure 18:**
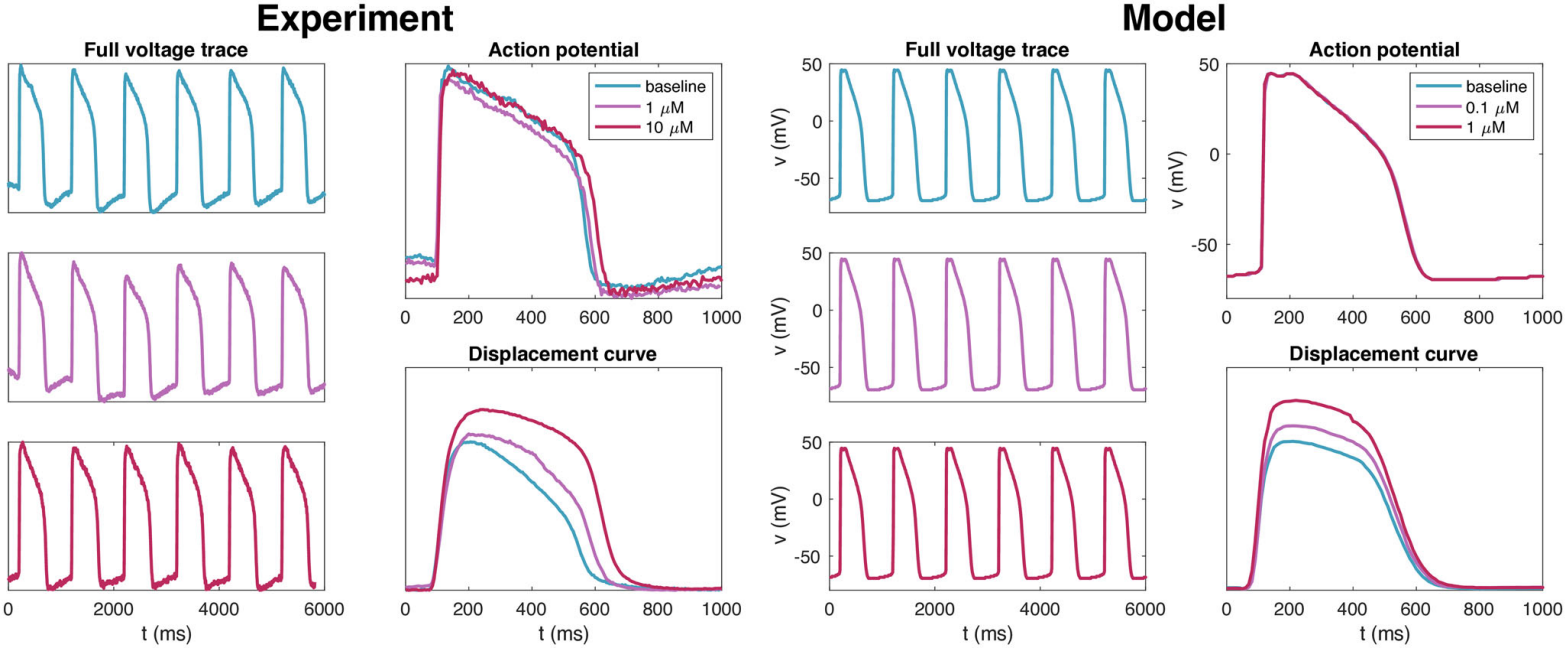
Omecamtiv. Full voltage traces, action potentials and displacement curves for a local area of Chip C7 in the baseline case and after application of 0.1 and 1 µM of omecamtiv. The left panels show optical measurements from an experiment, and the right panels show the model solution. The corresponding biomarker values are provided in Table 8. The effect of the drug is modelled as described in Table 9.

#### Summary of drug properties

The effects of each of the considered drugs in terms of changes in biophysical parameter values are summarized in Table 9. The table also reports corresponding parameter changes gathered from literature. When computing parameter changes from literature values, the dose‐dependent change in a parameter, p, is assumed to be given on the form

(38) $$p\left(d\right)=\left(1+\frac{E_{\max}}{1+{\left(\frac{{\mathrm{EC}}_{50}}{d}\right)}^{h}}\right)p\left(0\right),$$

where p(d) is the parameter value for the drug dose d, p(0) is parameter value in the absence of drugs, $E_{\max}$ is the maximal effect of the drug (e.g., $E_{\max}=-1$ for a complete channel blocker), h is a Hill coefficient and EC_50_ is the half maximal effective concentration of the drug. In cases where literature only provides EC_50_, h is assumed to be 1 and $E_{\max}$ is assumed to be −1.

## Discussion

### Measured and computed effects of drugs

As listed in Table 9, we have analysed the effects of six drugs: flecainide, quinidine, nifedipine, verapamil, blebbistatin and omecamtiv. The effects of these drugs at two different doses were compared with baseline biomarker values. For each drug, we adjusted the model parameters to best replicate the experimental results, minimizing the differences between measured and computed biomarkers. The resulting parameter adjustments are detailed in Table 9, where they are compared to values reported in the literature. Whereas literature values show considerable variability, our results consistently fall within or fairly close to the reported ranges. This indicates that the combination of MPS measurements and computational modelling can be used to identify drug effects also for spatially resolved data.

Spatially resolved data have been previously used to identify drug effects, notably with the bidomain model combined with measurements of extracellular potentials using microelectrode arrays (MEA); see Asakura et al. (2015), Bouyssier and Zemzemi (2017), Clements and Thomas (2014), Jæger, Charwat et al. (2021), Tixier et al. (2018) and Zwi et al. (2009). However, this study represents the first application of spatially resolved cell‐based models to identify drug effects based on measurements of transmembrane potential and mechanical displacement.

### Local or global averages?

To assess perturbations to the transmembrane potential or the mechanical displacements using images, it is necessary to average the data over a number of pixels. When the cells are synchronized, average over the whole chip is reasonable. However, when a wave is travelling across the chip, local and global spatial averages become very different; see Fig. 7. For automatic analyses, it is important to distinguish between wave form data and synchronized data. Furthermore, synchronized electrophysiological data can be modelled using standard single‐cell models (see Fig. 8), which by far is the most used approach in the field, but for wave form data, spatial effects must be taken into account.

### Modelling the dynamics of the chip

Measurements of the transmembrane potential or mechanical displacements of hiPSC‐CMs are typically performed in small MPSs, with the data usually averaged over the entire chip. Consequently, standard models formulated in terms of systems of ODEs representing spatially averaged data have been successfully applied. However, when waves traverse the chip, spatial effects must be considered, necessitating the use of spatially resolved models. Spatial modelling of cardiomyocytes in tissue is generally based on the bidomain model or the monodomain model; see, for instance, Franzone et al. (2014) and Sundnes et al. (2007). Nevertheless, these models are founded on assumptions that may not be easily validated for cells within the chip. The irregular geometry defined by the cells, as illustrated in Figs 5 and 6, presents additional challenges because it is not straightforward to define a proper computational domain. The SKNM/KNM approach, (Jæger & Tveito, 2023b, 2023c), avoids these issues by directly modelling the graph of cells, bypassing the need for a continuous computational domain. Another advantage of these models is that they allow for the relatively straightforward modelling of the effect of changing cell shapes based on the displacements computed in the models. As observed in an example video (Supporting Information), the movements caused by displacements in the model appear to reasonably mimic the movements seen in the measured data.

The mathematical model applied here is founded on the following principles:

1. The electrophysiology of the single‐cell model is taken from Jæger et al. (2020). This model has been used in several papers, (Huebsch et al., 2022; Jæger, Charwat et al., 2021; Jæger, Edwards et al., 2021; Jæger et al., 2022), where the density of currents and fluxes has been adopted to the application under consideration. However, this part of the model can easily be replaced by other single‐cell electrophysiology models of hiPSC‐CMs, for example, those proposed in Botti et al. (2024), Forouzandehmehr et al. (2021) and Kernik et al. (2019).
2. Here, the single‐cell model has been extended to account for mechanical contraction using a simplified representation of the commonly applied contraction model proposed in Rice et al. (2008). The contraction model is used to represent the altering of cell length as the cells contract during the action potential.
3. Each cell is represented as a 3D geometrical object, and the volume of each cell is assumed to be constant during contraction. Thus, the width of the cell is changed according to eqn (28).
4. Neighbouring cells are assumed to be mechanically coupled and the coupling is assumed to satisfy Hooke's law and Newton's second law.
5. Neighbouring cells are also electrically coupled based on Kirchhoff's current law leading to the KNM, see Jæger and Tveito (2023b). Here, we have applied the simplified SKNM version of the model which is exceptionally computationally efficient; see Jæger and Tveito (2023c).

### Synchronized transients are simpler computationally

Synchronized data in conjunction with computational models have repeatedly been used to successfully identify drug effects on hiPSC‐CMs, see for example, Jæger, Charwat et al. (2021), Kernik et al. (2019), Oslandsbotn et al. (2023), Paci et al. (2020), Paci et al. (2015) and Tveito et al. (2018). This approach has also been used to understand a very peculiar effect of the L‐type calcium channel blocker nifedipine that appears to increase the expression of L‐type calcium channels; see Jæger et al. (2024a). From a computational perspective, the single‐cell model, consisting of a straightforward system of ODEs, is significantly simpler to manage compared to spatially resolved models. The latter essentially requires solving a similarly complex system of ODEs for each cell in the chip. However, as observed in Fig. 9, perturbations to the cell length shortening in the single‐cell model do not necessarily match the perturbations to the displacement in the spatial model, indicating that spatial modelling might be required to accurately represent the mechanics of synchronous cell collections.

### Shortened diastolic interval for L‐type calcium channel blockers

From Figs 15 and 16 and Tables 5 and 6, we observe that application of the L‐type calcium channel blockers nifedipine and verapamil leads to a significant reduction in the diastolic interval, which aligns with the associated reductions in APD and the increase in BR. The effects observed in the experimental data and the model appear to be comparable. Numerical experiments indicate that the L‐type calcium channel block induces notable changes in ionic concentrations and membrane currents during the diastolic period, resulting in an elevated minimum membrane potential and a shorter diastolic interval.

### Measuring mechanical displacement

Inversion of the optical flow equation is considered the gold standard of evaluating mechanical displacements based on images, see, for example, Borzì et al. (2002) and Huebsch et al. (2015). The underlying assumption is that

(39) I(x+Δx,y+Δy,t+Δt)=I(x,y,t),

where I is the image, and Δx and Δy represent the displacement during the time step Δt. Classically, the Taylor series is used to assert that

(40) $$\begin{array}{ccc} & & I\left(x+\Delta x,y+\Delta y,t+\Delta t\right)=I+\Delta t\frac{\partial I}{\partial t}+\Delta x\frac{\partial I}{\partial x}\\ & & \;+\;\Delta y\frac{\partial I}{\partial y}+\text{higher-order terms}. \end{array}$$

By letting u=Δx/Δt and v=Δy/Δt, we get the optical flow equations in the limit of small step lengths,

(41) $$\frac{\partial I}{\partial t}+u\frac{\partial I}{\partial x}+v\frac{\partial I}{\partial y}=0.$$

Given a sequence of N images ${I(x,y,t_{i})}_{i=1}^{N}$, these equations can, in principle, be inverted to determine the velocity field (u,v) corresponding to the displacements. However, inverting partial differential equations is a challenging task, particularly when dealing with non‐smooth data. Also, for images, the non‐smooth data create very large higher‐order derivatives and therefore the removal of higher‐order terms may be questionable. To address these challenges, a simpler approach was developed in Sala et al. (2018) and is implemented in the Musclemotion software. This method involves directly comparing two images and calculating the difference in pixel values. For a detailed discussion of the properties of this method and a comparison with the inversion of the optical flow equation, see Sala et al. (2018). The approach proposed in Sala et al. (2018) is the same one used in eqn (1) above. In the figures and tables above, we observe good agreement in biomarker values between the measured data and the model results using eqn (1).

### Limitations

The experimental model applied here, (Charrez et al., 2021; Charwat et al., 2022; Huebsch et al., 2022), enables rapid, cost‐effective proarrhythmia risk prediction but has known limitations. hiPSC‐derived cardiomyocytes are immature, retaining spontaneous beating unlike adult ventricular cells. However, efforts have been made to enhance the maturation of hiPSC‐CMs through the development of specialized culture media. These advanced media formulations aim to address the limitations of traditional methods, which often produce cardiomyocytes with immature characteristics that do not fully replicate adult heart cells (Huebsch et al., 2022). Other limitations include inherent tissue variability and insufficient temporal resolution to capture the rapid upstroke of action potentials accurately.

The computational model also has limitations. The applied simplified cell geometries and cell locations might not fully capture the 3D structure of the collection of cardiomyocytes, potentially affecting mechanical and spatial dynamics. In addition, ion channels and transporters might be missing or represented in an overly simplified manner in the single‐cell action potential model. The considerably simplified representation of mechanical forces and mechanical interactions between cells is also a potential limitation of the computational model. Furthermore, parameterization of the action potential model is known to be non‐unique, where biomarkers can remain unchanged despite variations in model parameters (Jæger et al., 2019; Prinz et al., 2004).

Moreover, although our model allows for incorporating cellular heterogeneity, we currently lack sufficient data to characterize individual cell variations. As a result, we have assumed homogeneity in cell properties and gap junction coupling across cells in this model. We have also not incorporated randomness in cellular events. As a result, we have not been able to capture the synchronization variability observed in Chip C2 without applying a specified stimulation current.

## Conclusion

The use of hiPSC‐CMs represents a highly promising approach for studying the dynamics of human cardiomyocytes and their responses to pharmacological agents. However, significant advancements are still required to transform this approach into a stable, reliable technology capable of delivering reproducible results in a fully automated manner. In this study, we have shown that incorporating the wave properties of these cells is crucial for obtaining accurate representation of nonsynchronous data. Additionally, we have integrated mechanical deformation into our mathematical model, demonstrating that the computed displacements align well with experimental observations, as do other relevant biomarkers.

## Additional information

## Competing interests

All authors have financial relationships with Organos Inc.

## Author contributions

K.H.J. and A.T. developed the computational methodology and designed the numerical experiments. K.H.J. wrote the simulation code, performed the simulations and created the figures. A.T. conceived the project. S.W. and V.C. performed measurements of drug effects on hiPSC‐CMs. K.E.H. supervised experimental procedures. K.H.J., A.T., V.C. and S.W. wrote the main manuscript text. All authors approved the final version of the manuscript and agree to be accountable for all aspects of the work in ensuring that questions related to the accuracy or integrity of any part of the work are appropriately investigated and resolved. Furthermore, all persons designated as authors qualify for authorship, and all those who qualify for authorship are listed.

## Funding

This work was supported by the Research Council of Norway via INPART grant agreement #322312 (SIMBER) and by the European High‐Performance Computing Joint Undertaking EuroHPC under grant agreement #955495 (MICROCARD) co‐funded by the Horizon 2020 programme of the European Union (EU) and the Research Council of Norway.

## Supporting information


Peer Review History

**Baseline Parameters** Data set containing all model parameter values used for each of the different considered chips.

**Video 1** Example video file showing the depolarization wave in the baseline experiment for Chip C1 as well as the fitted model solution.

**Video 2** Example video file showing displacement from the baseline experiment for Chip C2 as well as the fitted model solution.

## Data Availability

The code and data used in our simulations are publicly available at Zenodo: https://doi.org/10.5281/zenodo.14191569.
