# Supplementary material for: Determining properties of human‐induced pluripotent stem cell‐derived cardiomyocytes using spatially resolved electromechanical metrics
Source: J Physiol. 2025 Feb 17;604(19):8069–99. doi: 10.1113/JP287275 (PMC13628578; doi:10.1113/JP287275)
Supplement: Supplementary file 1 [file TJP-604-8069-s002.pdf]

The Journal of Physiology

Determining Properties of Human Induced Pluripotent Stem Cell-Derived Cardiomyocytes Using Spatially Resolved Electromechanical Metrics

Karoline Horgmo Jæger, Verena Charwat, Kevin E. Healy, Samuel Wall, and Aslak Tveito
DOI: 10.1113/JP287275

Corresponding author(s): Karoline Horgmo Jæger (karolihj@simula.no)

The following individual(s) involved in review of this submission have agreed to reveal their identity: Stefano Severi (Referee #2)

Review Timeline:

Submission Date:	29-Aug-2024
Editorial Decision:	11-Nov-2024
Revision Received:	27-Nov-2024
Editorial Decision:	15-Jan-2025
Revision Received:	18-Jan-2025
Accepted:	28-Jan-2025

Senior Editor: Natalia Trayanova

Reviewing Editor: Christian Vergara

Transaction Report:

(Note: With the exception of the correction of typographical or spelling errors that could be a source of ambiguity, letters and reports are not edited. Depending on transfer agreements, referee reports obtained elsewhere may or may not be included in this compilation. Referee reports are anonymous unless the Referee chooses to sign their reports.)

Dear Dr Jæger,

Re: JP-RP-2024-287275 "Determining Properties of Human Induced Pluripotent Stem Cell-Derived Cardiomyocytes Using Spatially Resolved Electromechanical Metrics" by Karoline Horgmo Jæger, Verena Charwat, Kevin E. Healy, Samuel Wall, and Aslak Tveito

Thank you for submitting your manuscript to The Journal of Physiology. It has been assessed by a Reviewing Editor and by 2 expert referees and we are pleased to tell you that it is acceptable for publication following satisfactory revision.

The referee reports are copied at the end of this email.

Please address all the points raised and incorporate all requested revisions or explain in your Response to Referees why a change has not been made. We hope you will find the comments helpful and that you will be able to return your revised manuscript within 4 weeks. If you require longer than this, please contact journal staff: jp@physoc.org.

Your revised manuscript should be submitted online using the link in your Author Tasks Link Not Available. This link is accessible via your account as Corresponding Author; it is not available to your co-authors. If this presents a problem, please contact journal staff (jp@physoc.org). Image files from the previous version are retained on the system. Please ensure you replace or remove any files that are being revised.

If you do not wish to submit a revised version of your manuscript, you must inform our journal staff (jp@physoc.org) or reply to this email to request withdrawal. Please note that a manuscript must be formally withdrawn from the peer review process at one journal before it may be submitted to another journal.

TRANSPARENT PEER REVIEW POLICY: To improve the transparency of its peer review process The Journal of Physiology publishes online, as supporting information, the peer review history of all articles accepted for publication. Readers will have access to decision letters, including Editors' comments and referee reports, for each version of the manuscript, as well as any author responses to peer review comments. Referees can decide whether or not they wish to be named on the peer review history document.

ABSTRACT FIGURES: Authors are expected to use The Journal's premium BioRender account to create/redraw their Abstract Figures. Information on how to access this account is here: https://physoc.onlinelibrary.wiley.com/journal/14697793/biorender-access.

This will enable Authors to create and download high-resolution figures. If authors have used the free BioRender service, they can use the instructions provided in the link above to download a high-resolution version suitable for publication.
The link provided should only be used for the purposes of this submission. Authors will be charged for figures created on this account if they are not related to this manuscript submission.

LANGUAGE EDITING AND SUPPORT FOR PUBLICATION: If you would like help with English language editing, or other article preparation support, Wiley Editing Services offers expert help, including English Language Editing, as well as translation, manuscript formatting, and figure formatting at www.wileyauthors.com/eeo/preparation. You can also find resources for Preparing Your Article for general guidance about writing and preparing your manuscript at www.wileyauthors.com/eeo/prepresources.

RIGOUR AND REPRODUCIBILITY: We recommend authors consult The Journal's Rigour and Reproducibility requirements to ensure that their manuscript is suitable for publication: https://physoc.onlinelibrary.wiley.com/pb-assets/hub-assets/physoc/documents/TJP-Rigour-and-Reproducibility-Requirements.pdf

REVISION CHECKLIST:
Check that your Methods section conforms to journal policy: https://jp.msubmit.net/cgi-bin/main.plex?form_type=display_requirements#methods

Check that data presented conforms to the statistics policy: https://jp.msubmit.net/cgi-bin/main.plex?form_type=display_requirements#statistics

Upload a full Response to Referees file. To create your 'Response to Referees' copy all the reports, including any comments from the Senior and Reviewing Editors, into a Microsoft Word, or similar, file and respond to each point, using font or background colour to distinguish comments and responses and upload as the required file type.

Please upload two versions of your manuscript text: one with all relevant changes highlighted and one clean version with no changes tracked. The manuscript file should include all tables and figure legends, but each figure/graph should be uploaded as separate, high-resolution files. The journal is now integrated with Wiley's Image Checking service. For further details, see: https://www.wiley.com/en-us/network/publishing/research-publishing/trending-stories/upholding-image-integrity-wileys-image-screening-service

You may also upload:
- 'Potential Cover Art' for consideration as the issue's cover image
- Appropriate Supporting Information (Video, audio or data set: see https://jp.msubmit.net/cgi-bin/main.plex?form_type=display_requirements#supp).

We look forward to receiving your revised submission.

If you have any queries, please reply to this email and we will be pleased to advise.

Yours sincerely,

Natalia Trayanova
Senior Editor
The Journal of Physiology

REQUIRED ITEMS FOR REVISION

- Author photo and profile. First or joint first authors are asked to provide a short biography (no more than 100 words for one author or 150 words in total for joint first authors) and a portrait photograph. These should be uploaded and clearly labelled together in a Word document with the revised version of the manuscript. See Information for Authors for further details.

- The reference list must be in alphabetical order, rather than numbered, to comply with our Journal format.

- Please upload separate high-quality figure files via the submission form.

- Please ensure that the Article File you upload is a Word file.

- Your paper contains Supporting Information of a type that we no longer publish, including supplementary tables and figures. Any information essential to an understanding of the paper must be included as part of the main manuscript and figures. The only Supporting Information that we publish are video and audio, 3D structures, program codes and large data files. Your revised paper will be returned to you if it does not adhere to our Supporting Information Guidelines.

- Papers must comply with the Statistics Policy: https://jp.msubmit.net/cgi-bin/main.plex?form_type=display_requirements#statistics.

In summary:

- If n {less than or equal to} 30, all data points must be plotted in the figure in a way that reveals their range and distribution. A bar graph with data points overlaid, a box and whisker plot or a violin plot (preferably with data points included) are acceptable formats.

- If n > 30, then the entire raw dataset must be made available either as supporting information, or hosted on a not-for-profit repository, e.g. FigShare, with access details provided in the manuscript.

- 'n' clearly defined (e.g. x cells from y slices in z animals) in the Methods. Authors should be mindful of pseudoreplication.

- All relevant 'n' values must be clearly stated in the main text, figures and tables.

- The most appropriate summary statistic (e.g. mean or median and standard deviation) must be used. Standard Error of the Mean (SEM) alone is not permitted.

- Exact p values must be stated. Authors must not use 'greater than' or 'less than'. Exact p values must be stated to three

significant figures even when 'no statistical significance' is claimed.

- Please include an Abstract Figure file, as well as the Figure Legend text within the main article file. The Abstract Figure is a piece of artwork designed to give readers an immediate understanding of the research and should summarise the main conclusions. If possible, the image should be easily 'readable' from left to right or top to bottom. It should show the physiological relevance of the manuscript so readers can assess the importance and content of its findings. Abstract Figures should not merely recapitulate other figures in the manuscript. Please try to keep the diagram as simple as possible and without superfluous information that may distract from the main conclusion(s). Abstract Figures must be provided by authors no later than the revised manuscript stage and should be uploaded as a separate file during online submission labelled as File Type 'Abstract Figure'. Please also ensure that you include the figure legend in the main article file. All Abstract Figures should be created using BioRender. Authors should use The Journal's premium BioRender account to export high-resolution images. Details on how to use and access the premium account are included as part of this email.

- Please include a full title page as part of your main article (Word) file, which should contain the following: title, authors, affiliations, corresponding author name and contact details, keywords, and running title.

EDITOR COMMENTS

Reviewing Editor:

The Reviewers have finished their review process and sent their reports. Both encourage the authors to fix the reported points. Please prepare a Response to Referees with detailed answers and modify the text highlighting in colour your changes.

REFEREE COMMENTS

Referee #1:

The manuscript presents a new cell based electromechanical model for aggregates of human induced pluripotent stem cell-derived cardiomyocytes. After describing the model, the manuscript reports the results of several numerical experiments on different drug application scenarios. The simulated parameters have been compared critically with the experimental ones. The agreement between experiments and in-silico analysis is very good.

Overall the manuscript is very well written and organized. The results are original and interesting for the community. In summary, this is a very nice piece of work. Thus, I recommend publication after a minor revision according to the comments below.

1) What is the rationale of adopting 80% of repolarization to compute the action potential duration? The most popular choices in the literature are maximal slope or 90%.

2) What is the reason of loss of synchronicity in case of flecainide application? Is the model able to reproduce this behavior without stimulating artificially the leftmost or rightmost portion of the chip?

3) Among the papers on numerical solvers for cell based electrophysiology models, I suggest to add to the bibliography the following paper:

N. M. M. Huynh et al. Convergence analysis of BDDC preconditioners for composite DG discretizations of the cardiac cell-

by-cell model. SIAM J Sci Comput, 2023.

Referee #2:

Jaeger and colleagues present a fascinating computational study regarding the possible use of optical data from EHT experiments to estimate, using computational simulations, the drug-induced changes in the biophysical parameters of cardiomyocytes.

The work is relevant, novel, and well-presented. I have some comments and requests.

- The authors repeatedly claim that mono/bidomain models wouldn't be appropriate to run this kind of experiments. Can they produce evidence for this by showing the results of an illustrative case in which one of the simulations/estimations is performed with monodomain instead of SKNM?

- There are definitely too many references (40 to 51) for details on the EMI model. Please select one or two relevant papers to be referenced here.

- In the Methods section a short summary of the experimental data used in this work and their main characteristics should be given (e.g. resolution of images, number of experiments for each drug, etc etc) in the main text.

- The authors have included a model for cell contraction 'based on' the Ric one. Could you please summarize in the main text the main changes made with respect to that model?

- Section 2.2.2, subsection Electrophysiology: I would say Gi,j is the intercellular (rather than intracellular) conductance, please check.

- A limitation of the model to be acknowledged is the lack of heterogeneity between cells, given the very high level of heterogeneity in this kind of cells.

- The choice of modifying kXD to model Omecamtiv does not seem well justified. The experimental observations on which it is based are that the drug i) accelerates the step from D to X and ii) increases the time spent in bound states (X and P). In my view, the first observation should lead to an increase in both kDX and kXD (not only in kXD), whereas the latter should lead to a decrease in kPD (and, in case, also in kXP)

- Figure 6: was the EHT in Chip C1 self-oscillating? Simulated AP looks to have a completely stable resting potential with no self-depolarization, is this a characteristic of the IPS-CM ionic model?

- An illustrative video of the non-synchronous exp and sim chip would be helpful

- The increase in BR upon calcium current block is quite unexpected to me (it seems it is not entirely due to APD shortening, a concomitant, unexpected, shortening of the 'diastolic' phase is also present). Can you use the model to investigate the mechanism underlying this?

- It might be interesting to see the effects of the same simulated drug effects on single-cell simulations. Would the results be the same (in terms of APD, BR, and Cell shortening) as those measured in MPS? In particular, the relationship between DA and cell shortening would be nice to be explored.

- How was the tuning of parameters to reproduce drug effects performed? The authors write that differences between measured and computed biomarkers were 'minimized'. How was minimization ensured? I guess the authors cannot claim these are unique solutions, if so this should be acknowledged as a limitation section (by the way, the lack of a limitation subsection in a scientific paper is a bit disappointing).

- Refs #28 and #38 seem to be the same, please check for other duplications.

END OF COMMENTS

Response to Referees

Referee #1:

The manuscript presents a new cell based electromechanical model for aggregates of human induced pluripotent stem cell-derived cardiomyocytes. After describing the model, the manuscript reports the results of several numerical experiments on different drug application scenarios. The simulated parameters have been compared critically with the experimental ones. The agreement between experiments and in-silico analysis is very good.

Overall the manuscript is very well written and organized. The results are original and interesting for the community. In summary, this is a very nice piece of work. Thus, I recommend publication after a minor revision according to the comments below.

Authors' response:
Thank you very much for your review.

Referee #1:
1) What is the rationale of adopting 80% of repolarization to compute the action potential duration? The most popular choices in the literature are maximal slope or 90%.

Authors' response:
The reason why we use 80% repolarization to compute the action potential duration is that we have found this value to be most stable with respect to noise in the experimental measurements, while we have found, e.g., 90% repolarization to be more sensitive to noise in the data.

We have added the following text in Section 2.2.3 to clarify this point:

The choice of 80% repolarization aims to reduce the impact of random noise in the measurement data, which would be more pronounced at 90% repolarization.

Referee #1:
2) What is the reason of loss of synchronicity in case of flecainide application? Is the model able to reproduce this behavior without stimulating artificially the leftmost or rightmost portion of the chip?

Authors' response:
The loss of synchronicity in the case of flecainide application is likely due to flecainide's blocking of sodium channels, which leads to a reduction in the depolarizing sodium current across the cell membrane. One possible explanation for the apparent loss of synchrony is that an excitation wave may be present also in the baseline case, but that it propagates too fast to be detected at the current temporal resolution, giving the appearance of synchronous transients. When the sodium current is reduced by the application of flecainide, the

conduction velocity in the cell collection decreases, making the wave detectable. Another possibility is that the cells might beat in synchrony in the baseline case, but when the sodium current is removed, some cells lose their automaticity and only a few cells are still able to initiate the depolarization wave. Thus, the cell collection becomes non-synchronous after the application of flecainide. In this case, heterogeneity in cell properties within the cell collection and stochastic cellular events will determine where depolarization is initiated. Since depolarization is initiated at different location (left and right) for the two different doses of flecainide, the second possibility might be most likely. In order to reproduce this behavior in simulations, we would therefore have to incorporate randomness in cellular events and heterogeneity in cell properties. The modeling framework could be extended in this manner, but we believe it is beyond the scope of the current paper.

We address this point in the limitations section (Section 4.7) of the new version of the manuscript:

Moreover, although our model allows for incorporating cellular heterogeneity, we currently lack sufficient data to characterize individual cell variations. As a result, we have assumed homogeneity in cell properties and gap junction coupling across cells in this model. We have also not incorporated randomness in cellular events. As a result, we have not been able to capture the synchronization variability observed in Chip C2 without applying a specified stimulation current.

Referee #1:
3) Among the papers on numerical solvers for cell based electrophysiology models, I suggest to add to the bibliography the following paper:

N. M. M. Huynh et al. Convergence analysis of BDDC preconditioners for composite DG discretizations of the cardiac cell-by-cell model. SIAM J Sci Comput, 2023.

Authors' response:
We have now included this paper in the bibliography.

Referee #2:

Jaeger and colleagues present a fascinating computational study regarding the possible use of optical data from EHT experiments to estimate, using computational simulations, the drug-induced changes in the biophysical parameters of cardiomyocytes.

The work is relevant, novel, and well-presented. I have some comments and requests.

Authors' response:
Thank you very much for your review.

Referee #2:
The authors repeatedly claim that mono/bidomain models wouldn't be appropriate to run this kind of experiments. Can they produce evidence for this by showing the results of an illustrative case in which one of the simulations/estimations is performed with monodomain instead of SKNM?

Authors' response:
Monodomain and bidomain models are defined as partial differential equations (PDEs) operating on a continuous computational domain, which is discretized using a mesh. However, when cells are scattered in an irregular manner, it becomes challenging to define such a computational domain. For instance, constructing a domain requires identifying well-defined boundaries and a continuous and regular medium to represent the tissue, which is not straightforward when the spatial arrangement of cells is disordered. While one could theoretically approximate the cell arrangement using a density function or other heuristics, this introduces additional assumptions and uncertainties, which are not present in the SKNM/KNM approach.

In contrast, the SKNM/KNM models operate directly on a graph of coupled cells without requiring a continuous computational domain. This allows us to seamlessly transition from experimental images to a graph representation that is well-suited for simulations. However, such a graph is insufficient to define a proper computational domain for monodomain/bidomain models, highlighting their limitations for this type of analysis.

This limitation is noted in the manuscript, with the following updated text (new addition in red):

Spatial modeling of cardiomyocytes in tissue is generally based on the bidomain model or the monodomain model; see, for instance, [18, 72]. Nevertheless, these models are founded on assumptions that may not be easily validated for cells within the chip. The irregular geometry defined by the cells, as illustrated in Figure 5 and Figure 6 presents challenges because it is not straightforward to define a proper computational domain. The SKNM/KNM approach, [34, 35], avoids these issues by directly modeling the graph of cells, bypassing the need for a continuous computational domain.

Referee #2:
There are definitely too many references (40 to 51) for details on the EMI model. Please select one or two relevant papers to be referenced here.

Authors' response:
We have removed five references, but added one on the request of Referee #1.

Referee #2:
In the Methods section a short summary of the experimental data used in this work and their main characteristics should be given (e.g. resolution of images, number of experiments for each drug, etc etc) in the main text.

Authors' response:
In the original version of the manuscript, the information about the experimental methods was placed in the Supplementary Information. However, due to the editorial policies regarding the Supplementary Information, the experimental methods are now included in the main text.

Referee #2:
The authors have included a model for cell contraction 'based on' the Ric one. Could you please summarize in the main text the main changes made with respect to that model?

Authors' response:
Our model for cell contraction is now fully described in the main text.

Referee #2:
Section 2.2.2, subsection Electrophysiology: I would say Gi,j is the intercellular (rather than intracellular) conductance, please check.

Authors' response:
Yes, intracellular is misleading. The definition of G(i,j) is now moved from the Supplementary Information to the main text, and it is now referred to as intercellular.

Referee #2:
A limitation of the model to be acknowledged is the lack of heterogeneity between cells, given the very high level of heterogeneity in this kind of cells.

Authors' response:
We agree to this and add the following comment in the new limitations section in the manuscript:
Moreover, although our model allows for incorporating cellular heterogeneity, we currently lack sufficient data to characterize individual cell variations. As a result, we have assumed homogeneity in cell properties and gap junction coupling across cells in this model.

Referee #2:
The choice of modifying kXD to model Omecamtiv does not seem well justified. The experimental observations on which it is based are that the drug i) accelerates the step from D to X and ii) increases the time spent in bound states (X and P). In my view, the first observation should lead to an increase in both kDX and kXD (not only in kXD), whereas the latter should lead to a decrease in kPD (and, in case, also in kXP).

Authors' response:
We tried many ways of representing the effect of Omecamtiv and the representation used in the paper gave the best results in terms of fitting the model to the data. However, other parameter adjustments gave very similar results, and we agree with the reviewer that based on the mentioned observations from literature, other adjustments in addition to decreasing kXD would be suitable. In the new version of the manuscript, we now try to follow your suggestion: In order to accelerate the transition from D to X we have increased kDX. Furthermore, in order to increase the occupancy of P and X, we have reduced the transition rates from P to D and from X to D; i.e., we have reduced kPD and kXD. In order to maintain the lowest possible number of changes parameters, we have increased kDX, and reduced kPD, kXD by a multiplying a factor of (1+p) and (1-p), respectively.

Referee #2:
Figure 6: was the EHT in Chip C1 self-oscillating? Simulated AP looks to have a completely stable resting potential with no self-depolarization, is this a characteristic of the IPS-CM ionic model?

Authors' response:
Yes, the cells in Chip C1 are self-oscillating. The mathematical model can be either self-oscillating or reach a stable equilibrium based on the choice of parameters. For example, increasing the I_f current will lead to increase automaticity and so will a reduction of I_{K1}. In the current manuscript, all parameterizations of the model resulted in self-oscillating cells. However, for the model parameterization for Chip C1, the self-oscillating beat rate was slower than the beat rate used to stimulate the cells in the simulation.

Referee #2:
An illustrative video of the non-synchronous exp and sim chip would be helpful

Authors' response:
We have now included a video of the voltage in Chip C1 as a Supporting Information file.

Referee #2:
The increase in BR upon calcium current block is quite unexpected to me (it seems it is not entirely due to APD shortening, a concomitant, unexpected, shortening of the 'diastolic' phase is also present). Can you use the model to investigate the mechanism underlying this?

Authors' response:
Thank you for pointing this out, this is indeed interesting. We focused just on BR and APD, but clearly the diastolic interval is important and there is a significant perturbation that can be seen both in the experiments and in the model results. We have now run many simulations trying to understand what is going on and we have added the text below. We agree that this effect is unexpected and it may very well deserve further investigations, but it is beyond the scope of this paper to pursue this issue beyond a brief comment.

4.5 Shortened diastolic interval for L-type calcium channel blockers
From Figures 15 and 16 and Tables 5 and 6, we observe that application of the L-type calcium channel blockers Nifedipine and Verapamil leads to a significant reduction in the diastolic interval, which aligns with the associated reductions in APD and the increase in BR. The effects observed in the experimental data and the model appear to be comparable. Numerical experiments indicate that the L-type calcium channel block induces notable changes in ionic concentrations and membrane currents during the diastolic period, resulting in an elevated minimum membrane potential and a shorter diastolic interval.

Referee #2:
It might be interesting to see the effects of the same simulated drug effects on single-cell simulations. Would the results be the same (in terms of APD, BR, and Cell shortening) as those measured in MPS? In particular, the relationship between DA and cell shortening would be nice to be explored.

Authors' response:
We have now included a brief section in the manuscript including simulations of drug effects using the single cell model; see Section 3.1.3 and Figure 9.

Referee #2:
How was the tuning of parameters to reproduce drug effects performed? The authors write that differences between measured and computed biomarkers were 'minimized'. How was minimization ensured? I guess the authors cannot claim these are unique solutions, if so this should be acknowledged as a limitation section (by the way, the lack of a limitation subsection in a scientific paper is a bit disappointing).

Authors' response:
A subsection on limitations has now been added including a comment on the non-uniqueness of the parameters:

4.7 Limitations
The experimental model applied here, [22, 9, 10], enables rapid, cost-effective proarrhythmia risk prediction but has known limitations. hiPSC-derived cardiomyocytes are immature, retaining spontaneous beating unlike adult ventricular cells. However, efforts have been made to enhance the maturation of hiPSC-CMs through the development of specialized culture media. These advanced media formulations aim to address the limitations of traditional methods, which often produce cardiomyocytes with immature

characteristics that do not fully replicate adult heart cells [22]. Other limitations include inherent tissue variability and insufficient temporal resolution to capture the rapid upstroke of action potentials accurately.

The computational model also has limitations. The applied simplified cell geometries and cell locations might not fully capture the 3D structure of the collection of cardiomyocytes, potentially affecting mechanical and spatial dynamics. In addition, ion channels and transporters might be missing or represented in an overly simplified manner in the single cell action potential model. The considerably simplified representation of mechanical forces and mechanical interactions between cells is also a potential limitation of the computational model. Furthermore, parameterization of the action potential model is known to be non-unique, where biomarkers can remain unchanged despite variations in model parameters, [62, 39].

Moreover, although our model allows for incorporating cellular heterogeneity, we currently lack sufficient data to characterize individual cell variations. As a result, we have assumed homogeneity in cell properties and gap junction coupling across cells in this model. We have also not incorporated randomness in cellular events. As a result, we have not been able to capture the synchronization variability observed in Chip C2 without applying a specified stimulation current.

Referee #2:
Refs #28 and #38 seem to be the same, please check for other duplications.

Authors' response:
Thank you for pointing this out. We have now removed this and other duplicate reference entries.

Dear Dr Jæger,

Re: JP-RP-2024-287275R1 "Determining Properties of Human Induced Pluripotent Stem Cell-Derived Cardiomyocytes Using Spatially Resolved Electromechanical Metrics" by Karoline Horgmo Jæger, Verena Charwat, Kevin E. Healy, Samuel Wall, and Aslak Tveito

Thank you for submitting your manuscript to The Journal of Physiology. It has been assessed by a Reviewing Editor and by 2 expert referees and we are pleased to tell you that it is acceptable for publication following satisfactory revision.

The referee reports are copied at the end of this email.

Please address all the points raised and incorporate all requested revisions or explain in your Response to Referees why a change has not been made. We hope you will find the comments helpful and that you will be able to return your revised manuscript within 4 weeks. If you require longer than this, please contact journal staff: jp@physoc.org.

Your revised manuscript should be submitted online using the link in your Author Tasks Link Not Available. This link is accessible via your account as Corresponding Author; it is not available to your co-authors. If this presents a problem, please contact journal staff (jp@physoc.org). Image files from the previous version are retained on the system. Please ensure you replace or remove any files that are being revised.

If you do not wish to submit a revised version of your manuscript, you must inform our journal staff (jp@physoc.org) or reply to this email to request withdrawal. Please note that a manuscript must be formally withdrawn from the peer review process at one journal before it may be submitted to another journal.

TRANSPARENT PEER REVIEW POLICY: To improve the transparency of its peer review process The Journal of Physiology publishes online, as supporting information, the peer review history of all articles accepted for publication. Readers will have access to decision letters, including Editors' comments and referee reports, for each version of the manuscript, as well as any author responses to peer review comments. Referees can decide whether or not they wish to be named on the peer review history document.

ABSTRACT FIGURES: Authors are expected to use The Journal's premium BioRender account to create/redraw their Abstract Figures. Information on how to access this account is here: https://physoc.onlinelibrary.wiley.com/journal/14697793/biorender-access.

This will enable Authors to create and download high-resolution figures. If authors have used the free BioRender service, they can use the instructions provided in the link above to download a high-resolution version suitable for publication.
The link provided should only be used for the purposes of this submission. Authors will be charged for figures created on this account if they are not related to this manuscript submission.

LANGUAGE EDITING AND SUPPORT FOR PUBLICATION: If you would like help with English language editing, or other article preparation support, Wiley Editing Services offers expert help, including English Language Editing, as well as translation, manuscript formatting, and figure formatting at www.wileyauthors.com/eeo/preparation. You can also find resources for Preparing Your Article for general guidance about writing and preparing your manuscript at www.wileyauthors.com/eeo/prepresources.

RIGOUR AND REPRODUCIBILITY: We recommend authors consult The Journal's Rigour and Reproducibility requirements to ensure that their manuscript is suitable for publication: https://physoc.onlinelibrary.wiley.com/pb-assets/hub-assets/physoc/documents/TJP-Rigour-and-Reproducibility-Requirements.pdf

REVISION CHECKLIST:
Check that your Methods section conforms to journal policy: https://jp.msubmit.net/cgi-bin/main.plex?form_type=display_requirements#methods

Check that data presented conforms to the statistics policy: https://jp.msubmit.net/cgi-bin/main.plex?form_type=display_requirements#statistics

Upload a full Response to Referees file. To create your 'Response to Referees' copy all the reports, including any comments from the Senior and Reviewing Editors, into a Microsoft Word, or similar, file and respond to each point, using font or background colour to distinguish comments and responses and upload as the required file type.

Please upload two versions of your manuscript text: one with all relevant changes highlighted and one clean version with no changes tracked. The manuscript file should include all tables and figure legends, but each figure/graph should be uploaded as separate, high-resolution files. The journal is now integrated with Wiley's Image Checking service. For further details, see: https://www.wiley.com/en-us/network/publishing/research-publishing/trending-stories/upholding-image-integrity-wileys-image-screening-service

You may also upload:
- 'Potential Cover Art' for consideration as the issue's cover image
- Appropriate Supporting Information (Video, audio or data set: see https://jp.msubmit.net/cgi-bin/main.plex?form_type=display_requirements#supp).

We look forward to receiving your revised submission.

If you have any queries, please reply to this email and we will be pleased to advise.

Yours sincerely,

Natalia Trayanova
Senior Editor
The Journal of Physiology

EDITOR COMMENTS

Reviewing Editor:

The reference list must be in alphabetical order, rather than numbered, to comply with our Journal format.

Ethics Concerns:
The authors should clearly provide the following information in the manuscript:

- Informed consent for human data, preferably in writing

- Statement that the study is conformed to the standards set by the Declaration of Helsinki

- The procedures have been approved by the local ethics committee and the appropriate reference number has been stated

Dear Authors,

The Referees are satisfied with the revised version. There is no need for further changes from their side.

However, it seems that ethical and data information are missing (as above), please provide the required information.

REFEREE COMMENTS

Referee #1:

The manuscript is now suitable for publication in JP.

Referee #2:

The authors have significantly improved the manuscript and have satisfactorily addressed all my comments.

END OF COMMENTS

Response to editorial comments

EDITOR COMMENTS
Reviewing Editor:

The reference list must be in alphabetical order, rather than numbered, to comply with our Journal format.

Authors' response: The reference list is now in alphabetical order.

Ethics Concerns:
The authors should clearly provide the following information in the manuscript:

- Informed consent for human data, preferably in writing

- Statement that the study is conformed to the standards set by the Declaration of Helsinki

- The procedures have been approved by the local ethics committee and the appropriate reference number has been stated

Authors' response: There were no human subjects or human data used in our study. We have included the following Ethics section in the Methods section of the revised manuscript:

Ethics:
There was no ethics approval required for this study. Our general use of stem cell derived cardiomyocytes was assessed by the UC Berkeley Stem Cell Oversight Committee (SCRO), who determined that only notification of the use was required for the specific application. This notification was accepted with a SCRO reference number 2020-0-042.

Dear Authors,

The Referees are satisfied with the revised version. There is no need for further changes from their side.

However, it seems that ethical and data information are missing (as above), please provide the required information.

Authors' response: We have attempted to specify the ethical information as requested (see response above).

REFEREE COMMENTS

Referee #1:
The manuscript is now suitable for publication in JP.

Referee #2:
The authors have significantly improved the manuscript and have satisfactorily addressed all my comments.

2nd Revision - Editorial Decision **28-Jan-2025**

Dear Dr Jæger,

Re: JP-RP-2025-287275R2 "Determining Properties of Human Induced Pluripotent Stem Cell-Derived Cardiomyocytes Using Spatially Resolved Electromechanical Metrics" by Karoline Horgmo Jæger, Verena Charwat, Kevin E. Healy, Samuel Wall, and Aslak Tveito

We are pleased to tell you that your paper has been accepted for publication in The Journal of Physiology.

The last Word (or similar) version of the manuscript provided will be used by the Production Editor to prepare your proof. When this is ready you will receive an email containing a link to Wiley's Online Proofing System. The proof should be thoroughly checked and corrected as promptly as possible.

Authors should note that it is too late at this point to offer corrections prior to proofreading. Major corrections at proof stage, such as changes to figures, will be referred to the Editors for approval before they can be incorporated. Only minor changes, such as to style and consistency, should be made at proof stage. Changes that need to be made after proof stage will usually require a formal correction notice.

All queries at proof stage should be sent to: TJP@wiley.com.

Following copyediting and typesetting the accepted version will be published online.

Yours sincerely,

Natalia Trayanova
Senior Editor
The Journal of Physiology

IMPORTANT POINTS TO NOTE FOLLOWING YOUR PAPER'S ACCEPTANCE:

If you would like to receive our 'Research Roundup', a monthly newsletter highlighting the cutting-edge research published in The Physiological Society's family of journals (The Journal of Physiology, Experimental Physiology, Physiological Reports, The Journal of Nutritional Physiology and The Journal of Precision Medicine: Health and Disease), please click this link, fill in your name and email address and select 'Research Roundup':
https://www.physoc.org/journals-and-media/membernews

• TRANSPARENT PEER REVIEW POLICY: To improve the transparency of its peer review process, The Journal of Physiology publishes online as supporting information the peer review history of all articles accepted for publication. Readers will have access to decision letters, including Editors' comments and referee reports, for each version of the manuscript as well as any author responses to peer review comments. Referees can decide whether or not they wish to be named on the peer review history document.

• You can help your research get the attention it deserves! Check out Wiley's free Promotion Guide for best-practice recommendations for promoting your work at: www.wileyauthors.com/eeo/guide. You can learn more about Wiley Editing Services which offers professional video, design, and writing services to create shareable video abstracts, infographics, conference posters, lay summaries, and research news stories for your research at: www.wileyauthors.com/eeo/promotion.

• IMPORTANT NOTICE ABOUT OPEN ACCESS: To assist authors whose funding agencies mandate public access to published research findings sooner than 12 months after publication, The Journal of Physiology allows authors to pay an Open Access (OA) fee to have their papers made freely available immediately on publication.

The Corresponding Author will receive an email from Wiley with details on how to register or log-in to Wiley Authors Services where you will be able to place an order.

• You can check if your funder or institution has a Wiley Open Access Account here: https://authorservices.wiley.com/author-resources/Journal-Authors/licensing-and-open-access/open-access/author-compliance-tool.html.
